# School absenteeism as a risk factor for self-harm and suicidal ideation in children and adolescents: a systematic review and meta-analysis

**DOI:** 10.1007/s00787-019-01327-3

**Published:** 2019-04-15

**Authors:** Sophie Epstein, Emmert Roberts, Rosemary Sedgwick, Catherine Polling, Katie Finning, Tamsin Ford, Rina Dutta, Johnny Downs

**Affiliations:** 1 NIHR Maudsley Biomedical Research Centre, South London and Maudsley NHS Foundation Trust, London, UK; 2 South London and Maudsley NHS Foundation Trust, London, UK; 3 Department of Child and Adolescent Psychiatry, King’s College London, London, UK; 4 National Addiction Centre, King’s College London, London, UK; 5 Department of Psychological Medicine, King’s College London, London, UK; 6 University of Exeter Medical School, Exeter, UK

**Keywords:** Self-harm, Suicide, Child and adolescent mental health, School Mental Health, School attendance, Epidemiology

## Abstract

Self-harm and suicidal ideation in children and adolescents are common and are risk factors for completed suicide. Social exclusion, which can take many forms, increases the risk of self-harm and suicidal ideation. One important marker of social exclusion in young people is school absenteeism. Whether school absenteeism is associated with these adverse outcomes, and if so to what extent, remains unclear. To determine the association between school absenteeism and both self-harm (including completed suicide) and suicidal ideation in children and adolescents, we conducted a systematic review of observational studies. We conducted meta-analysis and report a narrative synthesis where this was not possible. Meta-analysis of cross-sectional studies showed that school absenteeism was associated with an increased risk of self-harm [pooled adjusted odds ratio (aOR) 1.37, 95% confidence interval 1.20–1.57, *P* = 0.01] and of suicidal ideation (pooled aOR 1.20, 95% CI 1.02–1.42, *P* = 0.03). A small number of studies showed that school absenteeism had a longitudinal association with both adverse outcomes. Heterogeneity in the exposure and outcome variables, study design and reporting was prominent and limited the extent to which it was appropriate to pool results. School absenteeism was associated with both self-harm and suicidal ideation in young people, but this evidence was derived from a small number of cross-sectional studies. Further research into the mechanisms of this association could help to inform self-harm and suicide prevention strategies at clinical, school and population levels.

## Introduction

Suicide remains the second most common cause of death in young people aged 10–24 years [1–3]. Self-harm, defined as any act of self-injury or self-poisoning, regardless of intent [4] is an important public health problem for young people in its own right, with a prevalence of more than one in ten adolescents worldwide [5], as well as being the strongest single risk factor for future completed suicide [6]. There are a number of established risk factors for self-harm and suicidal behaviour in young people, including mental disorders such as depression, anxiety, attention deficit and hyperactivity disorder (ADHD) and conduct disorder, drug and alcohol misuse and personality characteristics such as impulsivity. Social factors such as low socioeconomic status, adverse childhood experiences, family discord and bullying are also known to be risk factors [7–9]. Suicidal behaviours are more common in females, but completed suicide is more common in males [5]. Restricted educational achievement and the absence of a feeling of ‘school connectedness’ also seem to be related to self-harm [5, 10], but other educational factors, including school attendance, which may be particularly pertinent in this age group, are yet to be explored in any detail. This is surprising, given that schools collect substantial amounts of data about educational factors, such as attendance, attainment and exclusions.

Both the concepts of school attendance problems (SAP) and self-harm are complex, and are defined and conceptualised differently across settings and professional groups. A recent conceptual framework proposed by Heyne et al. describes SAP as a broad collection of difficulties which include school refusal (involving emotional distress associated with school attendance and no attempt by the young person to hide their absence from their parents), truancy (without permission from the school and usually also concealed from parents), school withdrawal (where the absence from school is driven by the parent) and school exclusion (where the absence is due to a decision made by the school) [11]. However, because SAP are defined and described differently between studies, constructs cannot always be mapped directly onto the above categories. This systematic review addresses the concept of ‘school absenteeism’, broadly defined as absence from school for any reason, apart from school exclusion.

Self-harm research conducted in the United Kingdom (UK) and Europe typically use the definition provided in the first paragraph [4]. In the USA, however, the concepts of non-suicidal self-injury (NSSI) and suicide attempts are often considered separately, with greater emphasis placed on the presence or absence of suicidal intent [12]. NSSI refers to self-cutting rather than other forms of self-harm such as self-poisoning by overdose of medication [13, 14]. This review uses the broader definition of self-harm, as in many cases, suicidal intent (particularly in young people) is difficult to determine [15]. In addition, all types of self-harm, regardless of method and including self-harm without suicidal intent, are associated with later completed suicide [16–18].

Social exclusion, in the form of lower socioeconomic status [5, 19] and poor social capital, is known to increase the risk of self-harm in adults [20]. School is central to the social world of the majority of young people, holding importance for their sense of connectedness to the community outside their family [21]. Absence from school represents social exclusion for the young person affected. It also increases the likelihood of poor educational attainment [22, 23] and as such potentially further social exclusion in the future [24]. In adults, the aspect of individual socioeconomic position most consistently and strongly associated with self-harm is lower educational attainment [19]. Poor school attendance has been found to be related to a range of adverse outcomes in children and young people including violence, injury, substance misuse and a number of mental health problems [25–27]. Poor school attendance can also arise as a consequence of multiple forms of adversity such as personal or parental mental disorder (both internalising and externalising problems), bullying or abuse [25, 27–29] which are also known to be associated with self-harm.

Schools often act as the de facto front line mental health service for young people, with many more young people contacting teachers than health services about their mental health [30, 31]. Both education professionals and young people suggest that self-harm should be a priority issue to address in schools [32, 33]. However, education professionals feel ill-equipped to address it [34]. The recent UK government Green Paper ‘Transforming Children and Young People’s Mental Health Provision’ [35] has laid out plans to develop a system of greater integration between the health and education sectors in the provision of mental health support for young people. It promotes schools playing a greater role in the identification, prevention and management of mental health problems, including self-harm. In view of these proposals, it is essential to develop an evidence base which can improve our understanding of markers of vulnerability to self-harm and suicidal behaviours and inform the development of school-based self-harm and suicide prevention interventions.

Despite the availability of school attendance data and the policy context, to our knowledge there have been no previous reviews which examine school absenteeism as a risk factor for self-harm in young people. This systematic review aims to answer the following question: Does school absenteeism increase the risk of self-harm and suicidal ideation in school-age children and adolescents?

## Methods

This review is reported according to the Preferred Reporting Items for Systematic Reviews and Meta-analyses (PRISMA) guidelines [36] and the Meta-analysis of Observational Studies in Epidemiology (MOOSE) checklist [37]. The protocol is published [38] and is registered on PROSPERO (ID CRD42018088608).

### Search strategy

We searched Medline, PsycINFO, Embase, the Education Resources Information Centre (ERIC), and the British Education Index (BEI) from 1 January 1990 until 6 June 2018. Studies published prior to this range were excluded because they are likely to be less relevant to the present day, due to rapidly changing social contexts and education systems. The search strategies were developed to include both keywords and thesaurus terms for the population (e.g. child*, adolescen*), exposure (e.g. school* adj attend*) and outcome (e.g. suicid*, self-harm*) of interest. For keyword searching, truncation and wildcards were used to allow for linguistic variations. Full search strategies can be found in online supplementary materials.

Database searching was supplemented by forward and backward citation chasing of included studies, hand searching of reference lists of existing systematic reviews on risk factors for self-harm and suicidal behaviour in young people and hand searching of the journal ‘Suicide and Life Threatening Behaviour’. A list of included papers was also sent to experts in the field who were asked whether they were aware of any studies which had been missed (see Supplementary Materials).

### Inclusion criteria

Peer-reviewed papers reporting quantitative observational studies published in English from 1990 onwards were considered for inclusion. Qualitative studies, book chapters, case reports, conference proceedings, dissertation abstracts and intervention studies (unless they were reporting a specific intervention to target poor school attendance) were excluded.

Studies were included if all participants were enrolled in school at the point of enrolment into the study. Studies were included only where school absenteeism was considered as the exposure and self-harm or suicidal ideation the outcome. Exceptions to this rule were univariate analyses from crosssectional studies which were included regardless of whether absenteeism was considered the exposure or the outcome, because the relationship can be interpreted in either direction. Included studies were required to include a comparison group. Studies where all outcomes were measured in adulthood were excluded.

### Exposure and outcome variables

The definition of self-harm used in this review included non-suicidal self-injury, suicide attempts, completed suicide and any acts of self-harm where the intent was unknown. ‘Suicidal ideation’ includes suicidal ideation/thoughts or suicide plans. In the “Results” section of this review, suicidal ideation and self-harm are considered in separate sections. Studies that used composite measures of suicidal ideation and acts of self-harm are included under self-harm.

The definition of school absenteeism used is any form of non-attendance at school amongst pupils enrolled in school, including school refusal, school phobia, truancy or longterm absence due to ill health. School ‘dropout’ or not being enrolled in school is qualitatively different from having a school place but attending less often than peers, so studies reporting these exposures were excluded. School exclusion was also considered a separate construct and therefore not included in this synthesis.

### Screening and data extraction

Titles and abstracts were exported to Endnote X8 and duplicates removed. The references were screened against inclusion criteria by two independent reviewers (SE and RS). Full texts were obtained and screened by these two reviewers. At each stage, uncertainties were initially discussed between the two reviewers and if necessary with a third reviewer (JD).

Data were extracted independently by two reviewers (SE and ER) using an agreed data extraction form (see Supplementary Materials). Where two studies reported data from the same cohort, we included the study with the larger sample size, or if samples were identical the study with the longest follow-up period.

### Quality assessment

Risk of bias within the included studies was assessed using a modified Newcastle–Ottawa Scale (NOS) [39]. The NOS is a commonly used scale for assessing risk of bias in case control and cohort studies with a published adaptation for cross-sectional studies [40]. Risk of bias assessment was carried out by two independent reviewers (SE and ER). Some items were adapted for the purpose of this research question (see Supplementary Materials) and further quality parameters were added: appropriate sample size, appropriate statistical tests and clarity of reporting of exposure and outcome variables. For cross-sectional studies, the quality assessment was out of a possible ten points. Scores of 0–4 were considered as a high risk of bias, 5–7 as moderate risk of bias and 8–10 as a low risk of bias. For cohort and case control studies, where the assessment was out of a possible 13 points, scores of 0–5 were considered high, 6–9 moderate and 10–13 low risk of bias, respectively.

### Analysis

Synthesis was conducted for the outcomes of suicidal ideation and self-harm separately. Where possible, odds ratios (OR) were extracted from papers, or calculated from raw data. Otherwise, measures of association or correlation such as correlation coefficients or Chi square results are reported as presented in the papers. We planned to conduct subgroup analyses by gender, age and ethnicity where sufficient results were available.

Meta-analysis was performed where the following criteria were met: at least two results were derived from studies of the same design, reporting on similar exposures and outcomes, using the same summary statistic and in comparable populations (i.e. there was not a different set of demographics in the populations, e.g. all male or all female). Exposures were combined regardless of the number of days’ absence, the period of time over which absence was measured or whether absence was with or without permission. Outcomes were combined in two groups. First, any type of suicidal ideation or plans and second, any form of self-harm including completed suicide. These were combined regardless of the period of time over which they were measured. As per Cochrane guidance [41], only adjusted effect estimates were included in the meta-analyses. Where more than one adjusted effect size result was reported within one study, the following hierarchy was used to determine which to include in the meta-analysis: (1) the most comparable exposure and outcome measures, (2) those which adjusted for the greatest number of relevant covariates, (3) the most conservative (for example, if results were reported for an exposure of different numbers of days absent, the lowest number of days was used). Random effects metaanalysis using inverse variance weighting was conducted using RevMan v5.3 software and a pooled summary effect size is reported, as well as an *I* ^2^ estimate of heterogeneity. Funnel plots were constructed to examine publication bias among the studies included in the meta-analysis. Sensitivity analyses were conducted by removing outliers from the meta-analyses and removing studies with less similar exposure and outcome variables.

## Results

### Identified studies

**Fig. 1:**
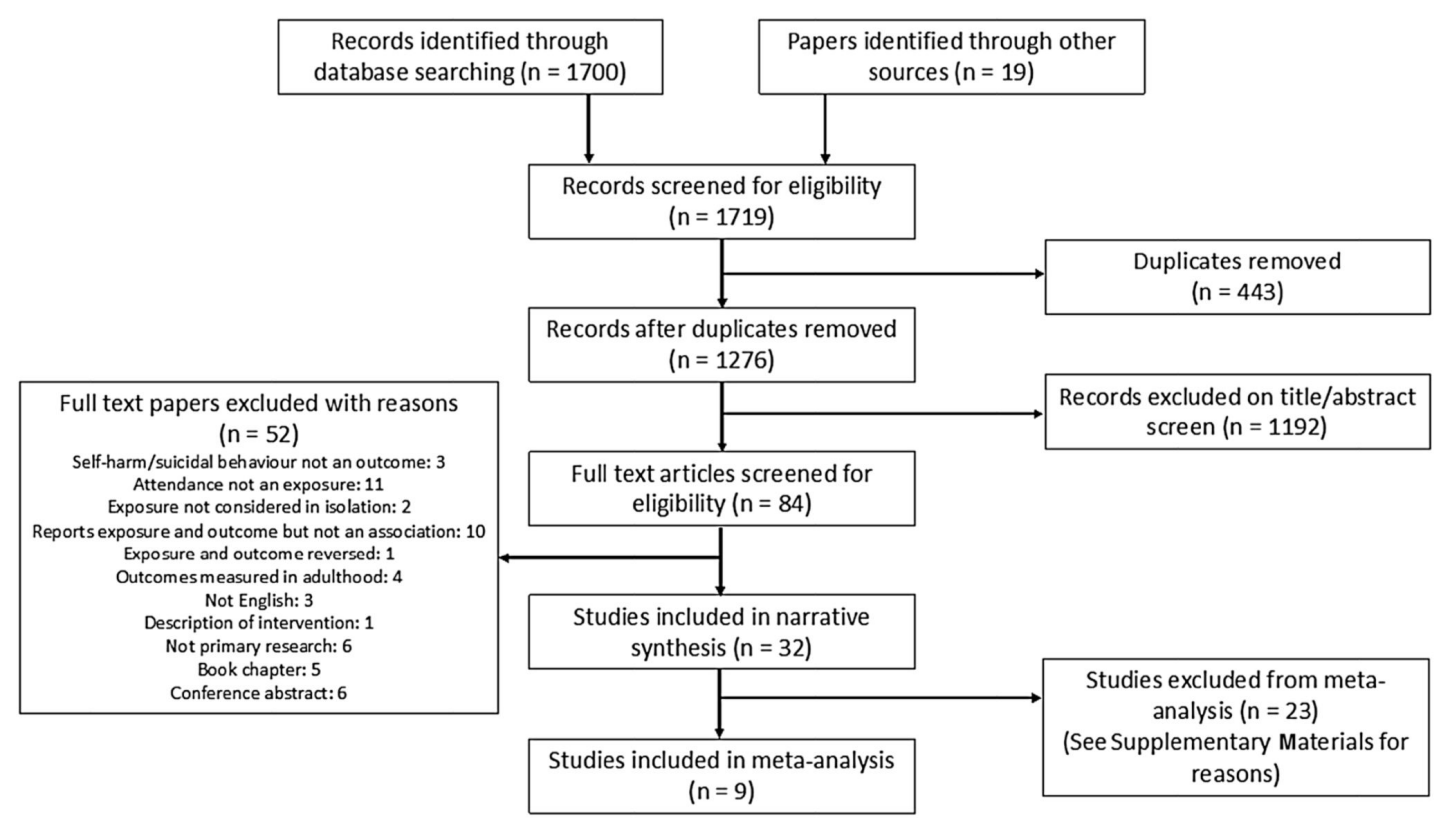
PRISMA flow diagram—selection of studies [36]

A total of 1700 references were identified through database searching and 19 from other search methods. 1276 remained after removing duplicates and 1192 were excluded on the title and abstract screen. 84 full texts were reviewed for inclusion and, of these, 32 were eligible for inclusion in the review. The PRISMA flow diagram below (Fig. 1) shows further details.

### Description of studies

Characteristics of all included studies can be found in Table 1. Each paper reports data from a unique population with the exception of Lewinsohn et al. [67] which reports longitudinal follow-up of the cross-sectional study reported by Lewinsohn et al. [66].

Of the 32 studies, 25 were cross-sectional, 5 were prospective cohort studies and 2 were case control studies. The mean or median age of participants in most studies was between 14 and 17 years. Most studies had a roughly equal proportion of male and female participants. All were conducted in general school populations, apart from two which studied American Indian and Alaskan native populations specifically [42, 43] (one of these studies compared those with single to those with multiple suicide attempts) [42], one conducted in a clinical African American population [44] and one which studied a population recently exposed to a natural disaster [45]. Studies were conducted in a range of high, middle and low income countries. Sample sizes ranged from 71 to over 70,000.

### Quality of included studies

Quality assessment scores using the modified NOS can be found in Table 1. The five cohort studies scored between 9 and 10 out of a possible 13 points on the modified NOS and therefore were deemed to have a low to moderate risk of bias. The 25 cross-sectional studies had scores ranging from 3 to 9 out of a possible 10 points with four studies scoring 4 or less (high risk of bias) and 11 studies scoring 8 or more (low risk of bias). The two case control studies scored 9 and 10 out of 13 (low to moderate risk of bias).

In the majority of studies, school absenteeism measures were self-reported, leading to possible recall or reporting bias. Selection bias may also be particularly problematic as the majority of studies were conducted in schools, and those students who were absent on the date(s) of data collection were often missed with no attempt to return at a later date to include these students. This is particularly pertinent in terms of the research question being addressed here, since not including absent pupils in the sample has the potential to underestimate any association that exists.

The loss to follow-up in the prospective cohort studies was not always accounted for and non-random attrition could bias the results. In the case of the current research question, this is again particularly pertinent as loss to follow-up is likely to be highly related to poor school attendance.

### School absenteeism and suicidal ideation

Sixteen papers reported results where the outcome of interest was suicidal ideation (Table 2). Of note, the exact definition of this construct varied between studies both in terms of the nature (e.g. seriously considered suicide, made a suicide plan) and the duration (e.g. lifetime, past 12 months). Full details of exposure and outcome variable constructs can be found in Supplementary Materials.

Seven studies reported a statistically significant association between school absenteeism and suicidal ideation after adjusting for potential confounders [45–51]. A prospective cohort study conducted in New Zealand by Fergusson et al. reported a more than twofold increase in the odds of suicidal ideation between age 14 and 21 years for those who had been truant from school between the age of 11 and 15 years (adjusted odds ratio (aOR) 2.09, 95% confidence interval 1.59–2.69) [49]. The remainder were cross-sectional studies and reported aORs of between 1.10 and 1.53. Cheng et al. reported an aOR of 0.49 (95% confidence interval 0.28–0.84) for no absence when compared to 6 days absence in the past 30 days. The result was not significant for 1–5 days absence when compared to 6 days [51]. A study conducted in Malaysia found a marginally significant association (aOR 1.1, 95% confidence interval 1.0–1.2) in only an urban sample, but failed to detect any effect in a rural sample [47]. Another was conducted in a population of those recently exposed to a natural disaster (aOR 1.48, 95% confidence interval 1.05–2.09) [45]. A large US sample (*n* = 12,095), however, did not detect an association between missing school due to feeling unsafe and either suicidal thoughts or plans where only a multivariate analysis was conducted [52].

Five further cross-sectional studies reported a statistically significant association at the *P* < 0.05 level in univariate analyses [53–57], three of which subsequently adjusted for confounders, resulting in the association being explained by confounding in two of the three cases [55, 56]. One study conducted in the Seychelles found no association in univariate analysis [58].

Interestingly, results from three studies suggested an inverse association, where in certain groups those with school absenteeism were shown to be at lower risk of suicidal thoughts [57, 59, 60]. One of these, by Peltzer and Pengpid, was a study conducted in several Southeast Asian countries where for the male subgroup, the aOR was 0.67 (95% confidence interval 0.48–0.94) [57]. Adjusted estimates for each individual country are also reported in the paper; however, none of these reached statistical significance at the *P* < 0.05 level. A second study, in the USA, also found a stronger inverse association in the male subgroup [60] (males: OR 0.70, 99% confidence interval 0.51–0.95; females: OR 0.74, 99% confidence interval 0.56–0.97), although a conservative significance cutoff of < 0.0033 compared to other studies was used. The third study reported that school absenteeism, after adjusting for potential confounders, was associated with a reduced risk of suicidal ideation [59].

**Fig. 2:**
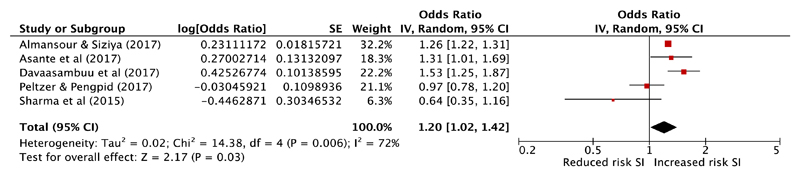
Meta-analysis of school absenteeism as a risk factor for suicidal ideation (SI). The study by Asante et al. reported effect estimates for both suicidal ideation and plans. The effect for suicide plans was included in this meta-analysis as it is the more conservative of the two

Multivariable results from five cross-sectional studies met criteria to be combined in meta-analysis [46, 48, 50, 55, 57] to provide a combined sample size of 42,233 (Fig. 2). All of these studies explored the relationship between suicidal ideation or plans and unauthorised absence from school. All had low (four studies) or moderate (one study) risk of bias. The pooled effect estimate demonstrates a 20% increase in odds of suicidal ideation in those with school absenteeism (pooled aOR 1.20, 95% confidence interval 1.02–1.42, *P* = 0.03). There was, however, a high level of heterogeneity between these studies with an *I* ^2^ of 72%. A funnel plot is included in the online Supplementary Materials, which demonstrates no evidence of publication bias (with one small negative study and no small positive studies). However, this should be interpreted with caution due to the small number of studies included. A sensitivity analysis removing this one small study with negative findings [55] results in a pooled aOR of 1.25 (95% confidence interval 1.08–1.45, P = 0.002, *I* ^2^ 68%).

### School absenteeism and self-harm

Twenty-four papers, which studied 23 separate samples, reported an outcome of self-harm (Table 3). Again, the outcome construct varied considerably between studies and included suicide attempts, completed suicide, self-harm behaviour and deliberate self-injurious behaviour. Durations of measurement ranged from 3 months to lifetime.

After adjusting for potential confounders, one prospective cohort study found a more than fourfold increased odds of suicide attempt by the age of 21 years in those who had been truant from school between the ages of 11 and 15 years (aOR 4.07, 95% confidence interval 2.45–6.74) [49]. A second cohort study found an increased risk of suicide attempts in those who had missed school, but only in certain gender and ethnic subgroups (Hispanic and white females and white males, but not in a black ethnicity subgroup) [61].

A positive association was also reported after adjustment for confounders in several cross-sectional studies. Davaasambuu et al. reported a 31% increased odds of suicide attempt in multivariable analysis (aOR 1.31, 95% confidence interval 1.03–1.67) [48]. Pillai et al. detected a strong relationship between missing four or more days of school in 3 months and a suicide attempt during the same period in Goa (India), but this relationship did not hold for lower levels of absence. This relationship was stronger when females were considered separately (7 or more days absent, aOR 5.9, 95% confidence interval 2.2–16.1) [62]. Epstein and Spirito report that missing school due to feeling unsafe was associated with an increased odds of suicide attempts in a US sample (aOR 1.78, 95% confidence interval 1.36–2.33) [52]. Donath et al. reported an increased odds of suicide attempts in those with a history of truancy (aOR 1.56, 95% confidence interval 1.50–1.69) [63] and Xin et al. reported an increased odds of non-suicidal self-injury in those with a history of truancy (aOR 1.4 95% confidence interval 1.16–1.69) [64]. Pages et al., in a community sample in France, found an increase in lifetime suicide attempt among boys who were ‘often absent from school’ (aOR 1.6, 95% confidence interval 1.03–3.20); however on calculation of a P value, this did not seem to be statistically significant to the *P* < 0.05 level [65]. Cheng et al. reported a reduced odds of suicide attempts in those who had not been absent from school in the past 30 days compared to those who had missed six or more days of school (aOR 0.35, 95% confidence interval 0.19–0.65) [51]. Finally, Lewinsohn et al. found a positive cross-sectional association [66] (aOR 1.4, 95% confidence interval 1.1–1.7), but when examining baseline school attendance with suicide attempts at 1 year followup, the association was no longer significant at the *P* < 0.05 level [67].

Several further studies found an association between acts of self-harm and missing school only on univariate analysis [43, 46, 56, 69–71], one of which is a prospective cohort study [70]. Of these, four conducted subsequent multivariate analysis, and on adjustment the association was explained by confounding factors [46, 56, 69, 71]. One study [55] in Peru found an inverse association, with reduced odds of suicide attempts in those who missed more than 3 days of school in 30 days (OR 0.52, 95% confidence interval 0.30–0.88); however, this again did not persist after adjusting for confounders. A second study, by Lyon et al., reported that absenteeism was associated with a significantly reduced risk (a 50-fold difference) of presenting to hospital with suicidal ideation. However, on calculating the unadjusted odds ratio for this study from the data available in the paper, we found this association was near to a twofold difference. As well as the statistical errors, the sample size, ascertainment biases and matching methodology made interpreting any results from this study problematic [44].

Some studies did not detect an association. Taliaferro et al. found no association between missing school and suicide attempts in either male or female samples in Minnesota; however, a univariate analysis was not conducted and more conservative significance cutoff (of *P* < 0.0033) was used in comparison to other studies [60]. Similarly, Noble et al. did not find an association between missing school due to feeling unsafe and non-suicidal self-injury in a case–control study where only multivariable analysis was conducted [72]. Bailey et al. reported a four times increased rate of completed suicide in those with a history of contempt of court for truancy however this was not statistically significant at the *P*< 0.05 level [73].Finally, a study in an American Indian population found no evidence of an association between multiple compared to single suicide attempts in those who missed more days of school due to feeling unsafe [42]. However, this study was also the only one comparing groups in both of which the young people had self-harmed.

**Fig. 3:**
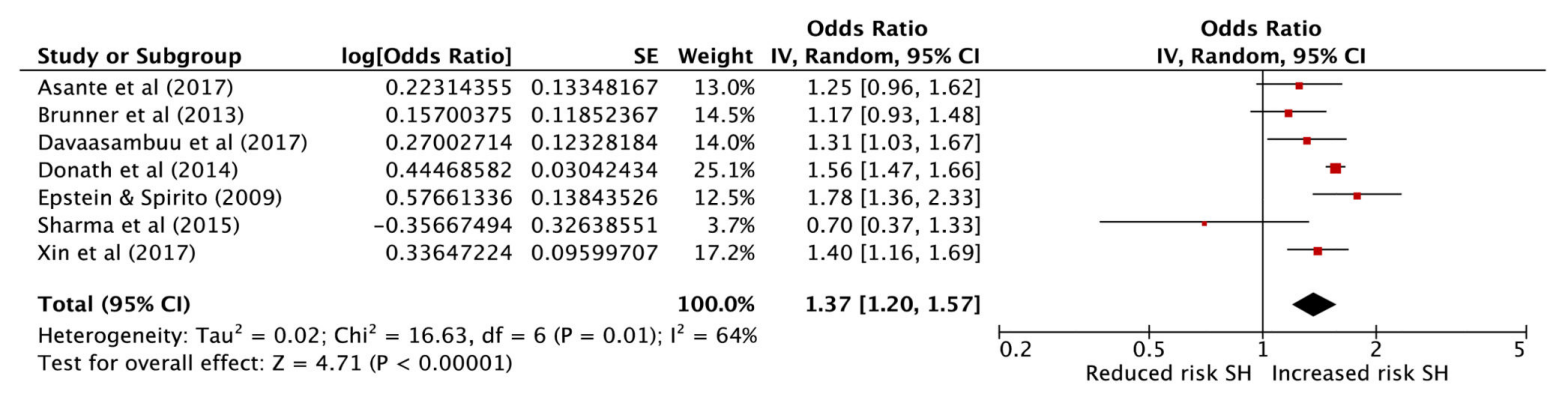
Meta-analysis of school absenteeism as a risk factor for self-harm (SH)

Multivariable results from seven cross-sectional studies met criteria for combination in a meta-analysis [46, 48, 52, 55, 63, 64, 69] to provide a combined sample size of 88,922 (Fig. 3). The exposure in all but one of these studies was unauthorised absence from school. The outcome was suicide attempts in five studies and deliberate self-injurious behaviour in two studies. All had low (five studies) or moderate risk (two studies) of bias. The pooled effect estimate demonstrates a 37% increase in odds of self-harm in those with school absenteeism (95% confidence interval 1.20–1.57, *P* = < 0.001). There was a moderate level of heterogeneity between these studies with an *I* ^2^ of 64%. A funnel plot is included in the online supplementary materials, which demonstrates no evidence of publication bias (with one small negative study and no small positive studies). However, this should again be interpreted with caution due to the small number of studies.

We conducted three sensitivity analyses as follows. First, removing the one small study with negative findings [55] resulted in a pooled aOR of 1.42 (95% confidence interval 1.26–1.59, *P* = <0.001, *I* ^2^ 55%). Second, removing the only study which did not specify that the absence was unauthorised [52] resulted in a pooled aOR of 1.32 (95% confidence interval 1.14–1.53, *P* = < 0.001, *I* ^2^ 67%). Finally, removing the two studies which report on deliberate self-injurious behaviour rather than suicide attempts [64, 69], resulted in a pooled aOR of 1.41 (95% confidence interval 1.18–1.67, *P* = < 0.001 *I* ^2^ 64%). In all three cases, the effect size and heterogeneity remained similar.

## Discussion

This systematic review provides evidence that school absenteeism is associated with both suicidal ideation and self-harm in young people. For both outcomes, although we found some studies that did not detect an association and some that reported an inverse association, when combinable effect estimates from multivariate analyses were pooled in meta-analyses, we detected a 20% increase in odds of suicidal ideation and a 37% increase in odds of self-harm for those with school absenteeism. As several of the individual studies contained in the meta-analyses did not report statistically significant results independently, a lack of statistical power could be an explanation for some of the studies finding no evidence of an association. The absence of a difference between groups in the single study which compared those with multiple compared to single suicide attempts suggests that although school absenteeism may be associated with the presence of self-harm behaviour, it may not be associated with an increase in severity or frequency [42]. This hypothesis would, however, require additional exploration.

Importantly, all of the studies included in the meta-analyses were cross-sectional, which means temporality and direction of a potential causal relationship cannot be determined. Only one study exploring suicidal ideation [49], one exploring completed suicide [73] and four exploring self-harm [49, 61, 67, 70] were longitudinal in design and the latter four could not be combined in meta-analysis. Nonetheless, these longitudinal studies do provide some evidence that school absenteeism acts as a risk factor for self-harm and suicidal ideation, which may occur through similar mechanisms as other forms of social exclusion such as low socioeconomic status, low social capital or a reduced sense of connectedness [19–21, 74, 75].

There are many possible mechanisms which could explain the relationship between school absenteeism and self-harm, each of which would require in-depth investigation, ideally through longitudinal and mixed methods research including the perspective of young people affected by these issues. The presence of mental disorder is one factor which could certainly play a role in this association, with depression, anxiety and externalising disorders known to be associated with both poor school attendance [25] and self-harm [7]. The presence of depression or anxiety could result in school absenteeism [11] and self-harm could form part of the presentation of these disorders. Bullying is another important potential explanatory factor, with existing evidence that bullying is an established cause of school absenteeism [29] and also an important risk factor for self-harm [8].

A small number of studies reported an inverse relationship, where absence from school was protective against suicidal ideation [57, 60] and self-harm [44, 55]. In the case of the studies reporting on an outcome of self-harm, in one study the result is reflected only in a univariate analysis and an association was no longer observed after adjusting for confounders [55]. The result reported in the other study was difficult to interpret due to methodological problems described above [44]. For those reporting on suicidal ideation, absence from school was measured over a 30-day period. It is therefore possible that through being absent, an acute school-related stressor, such as peer victimisation or academic pressure [5, 76–78], could be temporarily alleviated, leading to a reduction in suicidal thoughts in the short term. This effect, however, may not be sustained and it may be that individuals who are experiencing the cumulative effects of peer victimisation and school absence are at higher risk of self-harm over time. Social exclusion is likely to be experienced to a greater extent by young people who are persistently absent from school, which in turn may increase the risk of self-harm either directly, or through other more complex mechanisms, including through having an impact on academic attainment [22, 79–81].

## Strengths and limitations

This is the first systematic review exploring school absenteeism as a potential risk factor for self-harm and suicidal ideation in children and adolescents. We have used broad definitions of these constructs and brought together evidence from published observational studies in the international literature. We applied a robust search strategy, considered literature from both the health and education fields and used double screening and data extraction.

Although we were able to pool adjusted effect estimates from some studies, due to heterogeneity of included covariates, it was not possible to explore or understand possible causal mechanisms. In many cases, where a univariate association was found, this relationship did not persist after adjusting for confounders. From the studies covered in this review, we identified a number of factors, such as being unhappy, being exposed to bullying or victimisation, a lack of parental support and alcohol use, which showed strong associations with self-harm and suicidal ideation. It is unclear whether these factors are part of a causal pathway or act as confounders between school absenteeism and suicidal ideation or self-harm. With this in mind, several studies may have introduced overadjustment bias, which may have obscured the effect of absenteeism on these adverse outcomes [82].

We were unable to explore how different reasons for absence from school may impact on self-harm risk. Across the studies, poor school attendance was inconsistently defined and measured. This is a common and frequently recognised problem in the field of school attendance research [11, 83].

Understanding how absenteeism sub-types may have different paths to self-harm is important for selecting interventions. For example, absence from school without permission, or truancy, may increase young people’s risk of self-harm and suicidal behaviour [69] via exposure to externalising problems such as substance misuse [84]. In contrast, school refusal or excused absences may lead to self-harm via an internalising pathway [27, 28, 85]. However, theoretical pathways for the associations between internalising and externalising disorders, excused and unexcused absence patterns and adverse mental health outcomes, are far from being established. Recent research has demonstrated stronger associations between emotional disorders and unexcused absence compared to excused absence [86, 87]. Complicating matters further is the risk of circular reasoning that can occur in absence research. For example, the definition of school refusal often requires the presence of emotional difficulties and the absence of conduct problems [11]. We found that the strongest evidence for school absenteeism as a risk factor for self-harm came from a study which specified truancy as the exposure [48]. It is also important to note that all but one of the studies within our meta-analyses used unexcused absence as the exposure. In summary, due to limitations of evidence available, this review was not able to establish which absenteeism sub-type had a more or less pronounced effect on risk for self-harm and suicidality. This review highlights the need for a clearer consensus on absence definitions, and the need for future research to address the significant gaps in evidence on school absence aetiology and adverse outcome patterns.

Further limitations result from the nature of the included studies. In view of the heterogeneity of the studies in terms of study design, definitions of exposure and outcome variables and statistical methods, meta-analysis was necessarily limited and subgroup analyses could not be conducted. In terms of publication bias, funnel plots were constructed and statistical tests carried out (see Supplementary Materials); however, the results should be interpreted with caution due to the small number of studies contained in each meta-analysis. Publication bias is also made less likely by the fact that studies tended to report a large number of risk factors; a study with a negative result for school absenteeism is likely to have been published in any case, due to positive results for other factors.

Due to the broad nature of the studies, terms relating to school absenteeism were often not present in the title, abstract or keywords and so other relevant studies may have been missed from the database searches. Additionally, within the studies themselves, there is a risk of reporting bias because in some cases, effect estimates, confidence intervals and *p* values are reported only for those variables which were found to have statistically significant associations. Finally, this systematic review does not include foreign language papers or information from grey literature which could also add to the evidence base on this subject.

### Implications and further research

Even without an understanding of the direction of, or mechanisms which underlie the relationship between school absenteeism and self-harm or suicidal ideation, the observation that there is an association is important nonetheless. This could create the opportunity to use school absenteeism as a proxy marker for other, more difficult to measure factors which increase the risk of self-harm. In schools in most high income countries, attendance data are routinely collected and easily accessed, and could aid in early identification of those at increased risk. To add to this, further research into the mechanisms of this association could help to determine the most pertinent targets for intervention.

If issues can be identified at school, particularly using data which is routinely collected, this could help to inform strategies to intervene through addressing modifiable risks. This is particularly pertinent in the context of the UK Green Paper ‘Transforming Children and Young People’s Mental Health Provision’ [35], where school-based mental health provision is a major strategic priority. In more general terms, an integral role of the education system is to improve life opportunities, resilience and well-being of young people and, to achieve this, aims to support vulnerable and disadvantaged groups [88]. Understanding markers of vulnerability can help schools to achieve this aim.

Future research should explore the impact of SAP on self-harm and suicidal behaviour in adulthood. Although beyond the scope of this review, there have been studies which do report the presence of an association between school absence and suicidal behaviour in later life [89, 90]. A systematic review of such studies would be informative.

There are further complexities concerning the relationship between school absenteeism and self-harm that it would be useful to explore in order to inform interventions, such as whether improvements in school attendance over time serve to reduce self-harm in those already engaging in these behaviours. This could be explored using frameworks such as the Response to Intervention Model to Promote School Attendance and Decrease School Absenteeism developed by Kearney and Graczyk [91]. This approach uses a tiered system of universal early intervention through to intensive targeted intervention which is intended as a blueprint for education, mental health and other professionals to support young people with SAP [91]. This systematic review did not identify any studies exploring the relationship between improving attendance and self-harm, nor did it identify any randomised or non-randomised school attendance intervention studies reporting on self-harm or suicidal ideation as an outcome. This suggests that longitudinal studies of the relationship between school factors and self-harm over time and studies of school attendance interventions targeting self-harming behaviours would make useful contributions to future research.

## Conclusion

There is emerging evidence of an association between school absenteeism and both self-harm and suicidal ideation in children and adolescents which has the potential to inform suicide prevention strategies at clinical, school and population levels. There are, however, many questions which require further exploration, particularly through longitudinal studies to better understand the direction of the relationship, causal mechanisms and potential targets for intervention.

## Supplementary Material

**Electronic supplementary material** The online version of this article (https://doi.org/10.1007/s00787-019-01327-3) contains supplementary material, which is available to authorized users.

Supplementary material 1

## Figures and Tables

**Table 1:** Characteristics of the included papers

References	Country	Data collection	Study design	*N* (or case/controls)	% female	Age range at enrolment (mean/median)	Groups of covariates^A^	Quality assessment score
Almansour and Siziya [50]	Swaziland	2013	Cross-sectional	3680	51.2	< 11 to > 18	Age, gender, social, mental health, substances, family, behaviour	6/10
Asante et al. [46]	Ghana	2012	Cross-sectional	1973	45.7	< 14 to > 18	Age, mental health, behaviour, substances, family	8/10
Bailey et al. [73]	US	2006–10	Cohort	20,536/1,132,116^g^	NR	11–17	None	9/13
Bjarnason and Thorlindsson [71]	Iceland	1992	Cross-sectional	7018	50	10th grade (15.3)	School, social, family, substances, suicide suggestion	6/10
Borowsky et al. [43]	US^a^	NR	Cross-sectional	11,666	52	12–18	None	6/10
Borowsky et al. [61]	US	1994–6	Cohort	13,110	NR	7th–10th grade	Age, family, social	10/13
Brunner et al. [69]	11 European countries^b^	2009–10	Cross-sectional	12,068	55.7	(14.9)	Age, gender, social, family, mental health, behavioural, substances	8/10
Chen et al. [47]	Malaysia	2001	Cross-sectional	4500	53.6	12–19 (15.3)	Gender, school, substances, family, social, behavioural, mental health (only urban students in multivariate analysis)	6/10
Cheng et al. [51]	China	2003	Cross-sectional	9015	51	11–17	Age, gender, family, social	9/10
Choquet and Menke [53]	France	1988	Cross-sectional	1519	45	13–16	None	4/10
Cwik et al. [42]	US^c^	2006–2009	Cross-sectional	71	64.8	10–19 (16)	None	3/10
Davaasambuu et al. [48]	Mongolia	2013	Cross-sectional	5393	51.7	12–17	Gender, substances, behaviour, social, mental health	8/10
De Man et al. [59]	Canada	NR	Cross-sectional	558	51.3	11–18 (14)	Social, family, school, mental health, (unclear which were included in multivariate model)	5/10
Donath et al. [63]	Germany	2007–2008	Cross-sectional	44,610	48.7	9th grade (15.3)	Age, gender, family, social, substances, mental health	8/10
Epstein and Spirito [52]	US	2005	Cross-sectional	12,095	50	12 to 18+	Behaviour, social	6/10
Evren et al. [68]	Turkey	2012	Cross-sectional	4957	47.3	(15)	None	4/10
Fergusson et al. [49]	New Zealand	1992–1995	Cohort	1063	49.8	15–21	Not clear what is adjusted for	9/13
Kandel et al. [54]	US	1986	Cross-sectional	593	NR	9th and 11th grade	None	4/10
Larsson and Sund [70]	Norway	1998–2000	Cohort	2464	50.8	12–15 (13.9)	None	9/13
Lau et al. [45]	China^d^	2008	Cross-sectional	3324	55.7	61.7% < 15	Gender, age, social, impact of earth-quake, mental health, family	9/10
Lewinsohn et al. [66]	US	1987–1990	Cross-sectional	1710	52.9	14–18	Gender and then depression	7/10
Lewinsohn et al. [67]	US	1987–1989	Cohort	1508	NR	14–18	Gender	10/13
Lyon et al. [44]	US^e^	NR	Case control	38/76	82	12–17 (14.8)	Age, gender, social, mental health, substances	9/13
Noble et al. [72]	US	NR	Case control	638/638		11–19 (14.92)	Gender, age, social, behaviour, school factors	10/13
Pages et al. [65]	France	NR	Cross-sectional	11,718	51.9	11–21 (16.6)	Age (in logistic regression model: behaviour, mental health, substances)	7/10
Peltzer and Pengpid [57]	7 SE Asian countries^f^	2007–2013	Cross-sectional	30,284	51.2	13–15 (14.1)	Gender, age, social, mental health, substances, physical health	8/10
Pillai et al. [62]	India	2006	Cross-sectional	1594	51.4	16–25 (19.4)^h^	Gender, age, social, family	8/10
Randall et al. [56]	Benin	2009	Cross-sectional	2690	33.1	< 12 to 16+	Age, gender, mental health, social, substances	9/10
Sharma et al. [55]	Peru	2014	Cross-sectional	903	53.6	(15)	Family, social	8/10
Taliaferro and Muehlenkamp [60]	US	2010	Cross-sectional	70,722	50	9th and 12th grade	Age, ethnicity, social, family, behaviour, mental health, substances	8/10
Wilson et al. [58]	Republic of Seychelles	NR	Cross-sectional	1432	52	11–17 (14)	None	7/10
Xin et al. [64]	China	NR	Cross-sectional	11,880	50.5	10–20 (14.62)	Age, social	8/10

A Each group listed here may comprise several variables, *NR* not reported

a American Indian and Alaska native youth

b Austria, Estonia, France, Germany, Hungary, Ireland, Israel, Italy, Romania, Slovenia and Spain

c Apaches (American Indians) who attempted suicide within the past 90 days

d In Chengdu, China, after the Sichuan Earthquake

e African American population only, cases and controls taken from clinical population

f Cambodia, Indonesia, Malaysia, Myanmar, Philippines, Thailand and Vietnam

g Person years with truancy/person years without truancy (denominator is derived from the whole base population)

h Age 16–25 (19.4) years, but absence measured in younger subsample

**Table 2:** Results for school absenteeism and suicidal ideation

References	Suicidal ideation construct	School absenteeism construct	Subgroups	OR (unless otherwise specified)	95% confidence interval	*P* value	aOR^a^	95% confidence interval	*P* value
Almansour and Siziya [50]	Past 1 year suicidal ideation	Truancy (not defined)		**1.43**	**1.40–1.47**	**< 0.001** *	**1.26**	**1.22–1.31**	**< 0.001** *
Asante et al. [46]	Past 1 year suicidal ideation	Missed class without permission three or more times in the past 30 days		**1.52**	**1.20–1.92**	**< 0.001**	**1.40**	**1.06–1.84**	**< 0.05**
	Past 1 year suicidal plan			**1.39**	**1.11–1.73**	**< 0.001**	**1.31**	**1.01–1.69**	**< 0.05**
Chen et al. [47]	Seriously considered suicide in the past 12 months	Number of days absent from school in the past 30 days (continuous)	Urban	–	–	–	**1.1**	**1.0–1.2**	**0.046**
			Rural	–	–	–	–	–	NS^k^
Cheng et al. [51]	Seriously considered suicide in the past 12 months	6 or more days absent from school in the past 30 days		Ref	–	–	Ref	–	–
		1–5 days absent from school in the past 30 days		0.7^g^	0.36–1.36	0.2902	0.66	0.31–1.39	0.2742
		0 days absent from school in the past 30 days		**0.45** ^g^	**0.28–0.72**	**0.0011**	**0.49**	**0.28–0.84**	**0.0103**
Choquet and Menke [53]	Past 1 year suicidal ideation^b^	Absent from class in the past 12 months (unclear definition)		**2.13** *	**1.55–2.93** *	**0.0001** *	–	–	–
Davaasambuu et al. [48]	Suicide plan in the past 12 months	Missed class in the past 30 days without permission		**1.87**	**1.59–2.20**	**< 0.0001**	**1.53**	**1.25–1.87**	**< 0.0001**
			Urban	**1.98**	**1.55–2.53**	**< 0.0001**	**1.54**	**1.14–2.07**	**0.005**
			Rural	**1.85**	**1.48–2.31**	**< 0.0001**	**1.55**	**1.19–2.02**	**0.001**
De Man et al. [59]	Suicidal ideation on scale for suicidal ideation (continuous)	Absenteeism in terms of class periods absent (continuous)		*r* = 0.08^h^		> 0.001	–		Absenteeism reduces risk
Epstein and Spirito [52]	Considered suicide in the past 12 months	Skipped school because felt unsafe in the past 30 days		–	–	–	–	–	NS^k^
	Made suicide plan in the past 12 months			–	–	–	–	–	NS^k^
Fergusson et al. [49]	Suicidal ideation between age 14 and 21 years	Any truancy between age 11 and 15 years		–	–	–	**2.09** * ^i^	**1.59–2.69** *	**< 0.0001** *
Kandel et al. [54]	Suicidal ideation (composite measure of few weeks and 12 months) vs none^c^	Cuts class (ever vs never)		**1.74** *	**1.22–2.49** *	**0.0027** *	–	–	–
Lau et al. [45]	Suicidal ideation since earthquake 1 month ago	Absence from school since the earthquake when the school was not closed		**2**	**1.50–2.65** *	**< 0.001**	**1.48**	**1.05–2.09**	**< 0.05**
Peltzer and Pengpid [57]	Seriously considered suicide in the past 12 months	In the past 30 days, 1 or more days missed class without permission		**1.54**	**1.41–1.69** *	**0.0001** *	0.97	0.78–1.2	> 0.05
			Male	–	–	–	**0.67**	**0.48–0.94**	**< 0.05**
			Female	–	–	–	1.19	0.92–1.54	> 0.05
Randall et al. [56]	Seriously considered suicide in the past 12 months^d^	Missed 3 or more days without permission in the past 30 days		–	–	–	0.31^j^	0.04–2.64	0.261
	Made suicide plan in the past 12 months^d^			–	–	–	0.81^j^	0.42–1.55	0.492
	Considered suicide or made plan past 12 months^e^			**2.03** *	**1.36–3.03** *	**0.0007** *			
Sharma et al. [55]	Suicidal ideation in the past 12 months	3 or more days absent in the past 30 days without giving notice to the school		**2.07**	**1.28–3.36**	**< 0.01**	0.64	0.35–1.16	> 0.05
Taliaferro and Muehlenkamp [60]	Suicidal ideation in the past 12 months (and never suicide attempts)	Missed school due to feeling unsafe in the past 30 days	Male	–	–	–	**0.70**	**0.51–0.95**	**< 0.0033** ^l^
			Female	–	–	–	0.74	0.56–0.97	> 0.0033^l^
Wilson et al. [58]	Past 12 months, seriously considered attempting suicide or made a plan**^f^**	Missed 3 or more days in the past 30 days without permission		1.32*	0.84–1.85*	0.1322*	–	–	–

Data is missing where either univariate or multivariate analyses were not reported in the paper and could not be calculated from the data available

* Results calculated from data available within the paper; *OR* odds ratio, *NS* non-significant, significance cutoff not reported in paper; Variables are binary unless otherwise stated; statistically significant results in bold (using *P* < 0.05 cutoff unless otherwise stated)

a Adjusted for covariates listed in Table 1

b Dichotomised to never suicidal ideation vs rarely/often and male and female combined

c Dichotomised to suicidal ideation vs some/high and male and female combined

d Multinomial logistic regression with no ideation vs ideation only vs ideation + plans as outcomes

e Dichotomised to no ideation vs ideation or plan

f Combined suicidal ideation and planning

g Adjusted for age and city only

h *r* = correlation coefficient, one tailed *P* value < 0.001 considered as significance cutoff

i Converted from log odds ratio

j Relative risk ratio

k Result not reported in multivariate model due to non-significance

l NS > 0.0033 (Bonferroni correction)

**Table 3:** Results for school absenteeism and self-harm

References	Self-harm or suicidal acts construct	School absenteeism construct	Subgroups	OR (unless otherwise specified)	95% confidence interval	*P* value	aOR^a^	95% confidence interval	*P* value
Asante et al. [46]	Suicide attempts in the past 12 months	3 or more days absent in the past 30 days without permission		**1.52**	**1.22–1.90**	**< 0.001**	1.25	0.96–1.62	> 0.05
Bailey et al. [73]	Suicide deaths from official records	Contempt of court for truancy		4.1^o^	0.7–14.6	0.11	–	–	–
Bjarnason and Thorlindsson [71]	Suicide attempts ever	How often do you play truant from school^f^	Male	***R* = 0.25** ^p^	–	**< 0.001**	*R* = 0.00^p^	–	> 0.05
			Female	***R* = 0.22** ^p^	–	**< 0.001**	*R* = 0.00^p^	–	> 0.05
Borowsky et al. 1999 [43]	Suicide attempts ever	Skipped school in the past month		**1.94**	**1.75–2.16**	**< 0.0001**	–	–	–
Borowsky et al. [61]	Suicide attempt in the past 12 months (at 1 year follow-up)	This school year, how many times have you skipped school?^g^	Male: Black	–	–	–	–	–	> 0.05^u^
			Male: Hispanic	–	–	–	–	–	> 0.05^u^
			Male: White	–	–	–	**2.9**	–	**< 0.01**
			Female: Black	–	–	–	–	–	> 0.05^u^
			Female: Hispanic	–	–	–	**3.1**	–	**< 0.01**
			Female: White	–	–	–	**2.9**	–	**< 0.001**
Brunner et al. [69]	Any lifetime deliberate self-injurious behaviour	Missed 3 or more days in 2 weeks without permission		**2.59**	**2.14–3.13**	**< 0.01**	**1.17**	0.93–1.48	> 0.05
Cheng et al. [51]	During the past 12 months, how many times did you actually attempt suicide? (dichotomised)	6 or more days absent from school in the past 30 days		Ref	–	–	Ref	–	–
		1–5 days absent from school in the past 30 days		0.71^q^	0.32–1.55	0.3896	0.77	0.33–1.78	0.5440
		0 days absent from school in the past 30 days		**0.33** ^q^	**0.19–0.58**	**0.3896**	**0.35**	**0.19–0.65**	**0.0008**
Cwik et al. [42]	Multiple suicide attempts in the past 90 days (vs single attempt)	Days missed school because feared unsafe (past 30) 0 vs 1 or more^h^		1.06*	0.34–3.30*	0.919*	–	–	–
Davaasambuu et al. [48]	Suicide attempt in the past 12 months	Missed class in the past 30 days without permission		**1.94**	**1.60–2.34**	**< 0.0001**	**1.31**	**1.03–1.67**	**0.03**
			Urban	**2.35**	**1.78–3.11**	**< 0.0001**	**1.43**	**1.00–2.04**	**0.05**
			Rural	**1.70**	**1.31–2.22**	**< 0.0001**	1.22	0.87–1.71	0.24
Donath et al. [63]	Lifetime suicide attempt	Truancy ever		–	–	–	1.56	1.50–1.69	< 0.001
Epstein and Spirito [52]	Suicide attempts in the past 12 months	Skipped school because felt unsafe past 30 days		–	–	–	1.78	1.36–2.33	–
Evren et al. [68]	Self-harm behaviour within the past year	Truancy before age 13		**2.34**	**1.99–2.75**	**< 0.001**	–	–	–
		Absenteeism in days—none vs 1–14 or more than 15^i^		**1.74** *	**1.37–2.22** *	**0.0001** *	–	–	–
Fergusson et al. [49]	Suicide attempt between age 14 and 21	Any truancy between age 11 and 15		–	–	–	**4.07** *	**2.45–6.74** *	**< 0.0001** *
Larsson and Sund [70]	Self-harm or suicide attempt during 1 year follow-up^b^	Truancy within the last year, ordinal 1–4, none to more than once a month^j^		**2.56** *	**1.81–3.62** *	**0.0001** *	–	–	–
Lewinsohn et al. [66]	Suicide attempts past 12 months	Days missed school past 6 weeks^k^		**1.5**	1.2–1.8	**< 0.01**	**1.4**	**1.1–1.7**	**< 0.01**
Lewinsohn et al. [67]	Suicide attempts between baseline and 1 year follow-up	Missed school days in the past 6 weeks^l^		–	–	> 0.05*r*	–	–	NS^u^
Lyon et al. [44]	Presentation to hospital with suicide attempt	Truancy		**0.43** *	0.19–0.94*	0.054*	**51.3** ^s^	**5.35–490.2**	**0.0001**
Noble et al. [72]	Lifetime non-suicidal self-injury	Number of days missed school because felt unsafe past 30 days (ordinal)	Middle school	–	–	–	1.14	–	> 0.05
			High school	–	–	–	0.93	–	> 0.05
Pages et al. [65]	Lifetime suicide attempt	Often absent from school	Male	–	–	–	1.6	1.03–3.2	0.104*
				**2.48**	**2.14–2.87**	**< 0.0001**	–	–	–
Pillai et al. [62]	Suicidal ideation, plan or attempt in the past 3 months	1 or more days absent from school in the past 3 months^m^		**2.61** *	1.20–5.66*	0.0188*			
		1–3 days absent from school in the past 3 months^n^		–	–	–	1.4	0.5–4.0	> 0.05
		4–6 days absent from school in the past 3 months^n^		–	–	–	**3.0**	**1.1–7.7**	**< 0.05**
		7 or more days absent from school in the past 3 months^n^		–	–	–	**5.1**	**2.1–12**	**< 0.05**
Randall et al. [56]	One suicide attempt past 12 months^c^	Missed 3 or more days without permission in the past 30 days		–	–	–	0.79^t^	0.24–2.54	0.669
	2 or more suicide attempts past 12 months^c^			–	–	–	1.70^t^	0.61–4.74	0.290
	1 or more suicide attempts past 12 months^d^			**2.71** *	**1.79–4.10** *	**0.0001** *			
Sharma et al. [55]	Suicide attempts in the past 12 months	3 or more days absent in the past 30 days without giving notice to the school		**0.52**	**0.3–0.88**	**< 0.05**	0.7	0.37–1.33	0.228*
Taliaferro and Muehlenkamp [60]	Suicide attempt past 12 months^e^	During past 30 days, did you miss school due to feeling unsafe?	Male	–	–	–	1.06	0.64–1.74	> 0.0033^v^
	(compared to suicidal ideation only)		Male	–	–	–	1.23	0.82–1.85	> 0.0033^v^
	Suicide attempt past 12 months^e^		Female	–	–	–	1.06	0.68–1.66	> 0.0033^v^
	(compared to suicidal ideation only)		Female	–	–	–	1.23	0.89–1.71	> 0.0033^v^
Xin et al. [64]	Deliberate self-injurious behaviour in the past 12 months	Truancy (not defined)		–	–	–	**1.4**	**1.16–1.69**	**< 0.001**
			Male	**3.53**	**3.00–4.16**	**< 0.05**	**1.48**	**1.16–1.90**	**0.002**
			Female	**3.93**	**3.24–4.77**	**< 0.05**	–	–	NS^u^

Data is missing where either univariate or multivariate analyses were not reported in the paper and could not be calculated from the data available

* Results calculated from data available within the paper, *OR* odds ratio, *NS* non-significant, significance cutoff not reported in paper, Variables are binary unless otherwise stated. Statistically significant results in bold (using *P* < 0.05 cutoff unless otherwise stated)

a Adjusted for covariates listed in Table 1

b Dichotomised to self-harm or suicidal ideation vs no self-harm or suicidal ideation

c Multinomial logistic regression with no suicide attempts vs one suicide attempt vs two or more as outcomes

d Dichotomised to no attempts vs one or more attempts

e Compared to no suicidal ideation or attempts

f Ordinal: 1: never; 2 < monthly; 3 monthly; 4 weekly; 5 daily

g Unclear comparator: highest end of scale vs lowest end

h Dichotomised to 0 days missed vs 1 or more days missed

i Dichotomised to no absenteeism vs 1–14 or more than 15 days absent, not clear over what period measured

j Ordinal converted to binary but not clear where the split has been made

k Not clear what the comparator is—probably binary but not clear where the split has been made

l Continuous variable

m Dichotomised to 0 days absent vs 1 or more days

n Categorical exposure variable

o Rate ratio—person years until outcome

p Statistic ‘*R*’ is comparable to standardised beta coefficient in multiple regression

q Adjusted for age and city only

r Only Chi square analysis reported

s Number of times less likely to be a risk factor

t Relative risk ratio

u Result not reported due to non-significance

v NS > 0.0033 (Bonferroni correction)
