# Supplementary material for: School absenteeism as a risk factor for self-harm and suicidal ideation in children and adolescents: a systematic review and meta-analysis
Source: Eur Child Adolesc Psychiatry. 2019 Apr 15;29(9):1175–94. doi: 10.1007/s00787-019-01327-3 (PMC7116080; doi:10.1007/s00787-019-01327-3)
Supplement: Supplementary file 1 — Supplementary material 1 (PDF 485 kb) [file 787_2019_1327_MOESM1_ESM.pdf]

Supplementary materials

Poor school attendance as a risk factor for self-harm and suicidal ideation in children and adolescents: A systematic review and meta-analysis

Contents:

Page	
1	Search strategies
6	Experts consulted
7	Excluded papers with reasons
10	Data extraction
11	Exposure and outcome variables
15	Full list of covariates
16	Reasons for excluding from meta-analysis
17	Funnel plots for studies included in meta-analyses
18	Quality assessment tools
22	Results of quality assessment
30	PRISMA and MOOSE checklists

Search Strategies

PsycINFO (Ovid)

1. Child*.tw
2. Adolescen*.tw
3. Student*.tw
4. Exp Students/
5. Youth*.tw
6. Pupil*.tw
7. Schoolchild*.tw
8. (young adj (people or person).tw
9. teen*.tw
10. p?diatric.tw
11. exp Elementary School Students /
12. exp Suicide/
13. exp Suicide, Attempted/
14. exp Suicidal Ideation/
15. exp Drug Overdose/
16. exp Self-injurious Behaviour or exp Self Mutilation/
17. (autoaggress* or auto aggress* or automutilat* or cutt* or overdose* or (self adj2 cut*) or selfdestruct* or self destruct* or selfharm* or self harm* or selfimmolat* or self immolat* or selfinflict* or self inflict* or selfinjur* or self injur* or selfmutilat* or self mutilat* or selfpoison* or self poison* or suicid*).tw
18. exp school suspension/
19. exp school expulsion/
20. exp Educational Attainment Level/
21. (manage* adj move*).tw
22. (terminat* adj5 school*).tw
23. (exclusion or exclude*).tw

24. ‘failure to complete’.tw
25. expulsion.tw
26. suspension.tw
27. expel*.tw
28. ((school* or kindergarten or nursery or education) adj4 (attend* or non-attend* or refus* or absen* or school phobi* or truan*)).tw
29. truan*.tw
30. exp truancy/
31. exp school truancy/
32. exp School Attendance/
33. 1 or 2 or 3 or 4 or 5 or 6 or 7 or 8 or 9 or 10 or 11
34. 12 or 13 or 14 or 15 or 16 or 17
35. 18 or 19 or 20 or 21 or 22 or 23 or 24 or 25 or 26 or 27 or 28 or 29 or 30 or 31 or 32
36. 33 and 34 and 35

EMBASE (Ovid)

1. Child*.tw
2. Exp Child/
3. Adolescen*.tw
4. Exp Adolescent/
5. Student*.tw
6. Exp Students/
7. Youth*.tw
8. Pupil*.tw
9. Schoolchild*.tw
10. (young adj (people or person)).tw
11. teen*.ti,ab.
12. p?diatric*.ti,ab.
13. exp suicide/
14. exp Suicide, Attempted/
15. exp Suicidal ideation/
16. exp suicide attempt/
17. exp suicidal behavior/
18. exp Drug overdose/
19. exp Self-injurious behaviour or exp Self Mutilation/
20. (autoaggress$ or auto aggress$ or automutilat$ or auto mutilat$ or cutt$ or overdose$ or (self adj2 cut$) or selfdestruct$ or self destruct$ or selfharm$ or self harm$ or selfimmolat$ or self immolat$ or selfinflict$ or self inflict$ or selfinjur$ or self injur$ or selfmutilat$ or self mutilat$ or selfpoison$ or self poison$ or suicid$).ti,ab.
21. (expulsion adj5 school*).ti,ab
22. (Suspen* adj5 school*).ti,ab.
23. (manage* adj move*).ti,ab.
24. (Terminat* adj5 school*).ti,ab.
25. (expel* adj5 school*).ti,ab.
26. ((exclusion or exclude*) adj6 school*).ti,ab.
27. ("failure to complete" adj5 (school* or educat*)).ti,ab.
28. ((school* or kindergarten or nursery or education*) adj4 (attend* or non-attend* or refus* or absen* or school phobi* or truan*)).tw.
29. truan*.ti,ab
30. exp school attendance/
31. exp absenteeism/

32. exp truancy/
33. 1 or 2 or 3 or 4 or 5 or 6 or 7 or 8 or 9 or 10 or 11 or 12
34. 13 or 14 or 15 or 16 or 17 or 18 or 19 or 20
35. 21 or 22 or 23 or 24 or 25 or 26 or 27 or 28 or 29 or 30 or 31 or 32
36. 33 and 34 and 35

Medline (Ovid)

1. child*.tw.
2. exp Child/
3. adolescen*.tw.
4. exp Adolescent/
5. student*.tw.
6. exp Students/
7. youth*.tw.
8. pupil*.tw.
9. schoolchild*.tw.
10. (young adj (people or person)).tw.
11. teen*.ti,ab.
12. p?diatric*.ti,ab.
13. exp suicide/
14. exp Suicide, Attempted/
15. exp Suicidal Ideation/
16. exp Drug Overdose/
17. exp Self-Injurious Behavior/ or exp Self Mutilation/
18. (autoaggress$ or auto aggress$ or automutilat$ or auto mutilat$ or cutt$ or overdose$ or (self adj2 cut$) or selfdestruct$ or self destruct$ or selfharm$ or self harm$ or selfimmolat$ or self immolat$ or selfinflict$ or self inflict$ or selfinjur$ or self injur$ or selfmutilat$ or self mutilat$ or selfpoison$ or self poison$ or suicid$).ti,ab.
19. (expulsion adj5 school*).ti,ab.
20. (Suspen* adj5 school*).ti,ab.
21. (manage* adj move*).ti,ab.
22. Terminat* adj5 school*).ti,ab.
23. (expel* adj5 school*).ti,ab.
24. ((exclusion or exclude*) adj6 school*).ti,ab.
25. ("failure to complete" adj5 (school* or educat*)).ti,ab.
26. ((school* or kindergarten or nursery or education*) adj4 (attend* or non-attend* or refus* or absen* or school phobi* or truan*)).tw.
27. truan*.tw
28. exp absenteeism/
29. 1 or 2 or 3 or 4 or 5 or 6 or 7 or 8 or 9 or 10 or 11 or 12
30. 13 or 14 or 15 or 16 or 17 or 18
31. 19 or 20 or 21 or 22 or 23 or 24 or 25 or 26 or 27 or 28
32. 29 and 30 and 31

Education Resources Information Centre (ERIC) (EBSCO)

S50	S18 AND S46 AND S49
S49	S19 OR S20 OR S21 OR S22 OR S47 OR S48
S48	AB (autoaggress* OR “auto aggress*” OR automutilat* OR “auto mutilate*” OR cutt* OR overdos* OR (self N2 cut*) OR self-destruct* OR “self destruct*” OR selfharm* OR “self harm*” OR selfimmolat* OR “self immolate*” OR

	selfinflict* OR “self inflict*” OR selfinjur* OR “self injur*” OR selfmutilat* OR “self mutilate*” OR selfpoison* OR “self poison*” OR suicid*)
S47	AB (autoaggress* OR “auto aggress*” OR automutilat* OR “auto mutilate*” OR cutt* OR overdos* OR (self N2 cut*) OR self-destruct* OR “self destruct*” OR selfharm* OR “self harm*” OR selfimmolat* OR “self immolate*” OR selfinflict* OR “self inflict*” OR selfinjur* OR “self injur*” OR selfmutilat* OR “self mutilate*” OR selfpoison* OR “self poison*” OR suicid*)
S46	S23 OR S24 OR S25 OR S26 OR S27 OR S28 OR S29 OR S30 OR S31 OR S32 OR S33 OR S34 OR S35 OR S36 OR S37 OR S38 OR S39 OR S40 OR S41 OR S42 OR S43 OR S44 OR S45
S45	DE “Truancy” OR DE “Attendance” OR DE “Attendance Patterns”
S44	AB truan*
S43	TI truan*
S42	DE "Withdrawal (Education)"
S41	DE "Attendance" OR DE "Average Daily Attendance" OR DE "College Attendance"
S40	DE "Suspension"
S39	DE "Expulsion"
S38	AB expel*
S37	TI expel*
S36	AB suspension
S35	TI suspension
S34	AB expulsion
S33	TI expulsion
S32	AB "failure to complete"
S31	TI "failure to complete"
S30	AB (exclusion OR exclude*)
S29	TI (exclusion OR exclude*)
S28	AB (terminat* N5 school*)
S27	TI (terminat* N5 school*)
S26	AB (manage* N1 move*)
S25	TI (manage* N1 move*)
S24	AB ((school* OR kindergarten OR nursery OR education*) N4 (attend* OR non-attend* OR refus* OR absen* OR school phobi* OR truan* OR dropout*))
S23	TI ((school* OR kindergarten OR nursery OR education*) N4 (attend* OR non-attend* OR refus* OR absen* OR school phobi* OR truan* OR dropout*))
S22	DE "Self Destructive Behavior"
S21	AB suicid*
S20	TI suicid*
S19	DE "Suicide"
S18	S1 OR S2 OR S3 OR S4 OR S5 OR S6 OR S7 OR S8 OR S9 OR S10 OR S11 OR S12 OR S13 OR S14 OR S15 OR S16 OR S17
S17	AB (young N1 (people OR person))
S16	TI (young N1 (people OR person))
S15	AB schoolchild*
S14	TI schoolchild*

S13	AB pupil*
S12	TI pupil*
S11	AB youth*
S10	TI youth*
S9	DE "Students"
S8	AB student*
S7	TI student*
S6	DE "Adolescents"
S5	AB adolescen*
S4	TI adolescen*
S3	AB child*
S2	TI child*
S1	DE "Child Health" OR DE "Children"

British Education Index (BEI) (EBSCO)

S52	S20 AND S25 AND S51
S51	S26 OR S27 OR S28 OR S29 OR S30 OR S31 OR S32 OR S33 OR S34 OR S35 OR S36 OR S37 OR S38 OR S39 OR S40 OR S41 OR S42 OR S43 OR S44 OR S45 OR S46 OR S47 OR S48 OR S49 OR S50
S50	DE “School attendance” OR DE “School Absenteeism”
S49	AB truan*
S48	TI truan*
S47	DE "SCHOOL dropouts"
S46	DE "COLLEGE attendance"
S45	DE "SCHOOL attendance"
S44	DE "STUDENT suspension"
S43	DE "EXCLUSION from school"
S42	DE "STUDENT expulsion"
S41	AB expel*
S40	TI expel*
S39	AB suspension
S38	TI suspension
S37	AB expulsion
S36	TI expulsion
S35	AB "failure to complete"
S34	TI "failure to complete"
S33	AB (exclusion OR exclude*)
S32	TI (exclusion OR exclude*)
S31	AB (terminat* N5 school*)
S30	TI (terminat* N5 school*)

S29	AB (manage* N1 move*)
S28	TI (manage* N1 move*)
S27	AB ((school* OR kindergarten OR nursery OR education*) N4 (attend* OR non-attend* OR refus* OR absen* OR school phobi* OR truan* OR dropout*))
S26	TI ((school* OR kindergarten OR nursery OR education*) N4 (attend* OR non-attend* OR refus* OR absen* OR school phobi* OR truan* OR dropout*))
S25	S21 OR S22 OR S23 OR S24
S24	AB (autoaggress* OR “auto aggress*” OR automutilat* OR “auto mutilate*” OR cutt* OR overdos* OR (self N2 cut*) OR self-destruct* OR “self destruct*” OR selfharm* OR “self harm*” OR selfimmolat* OR “self immolate*” OR selfinflict* OR “self inflict*” OR selfinjur* OR “self injur*” OR selfmutilat* OR “self mutilate*” OR selfpoison* OR “self poison*” OR suicid*)
S23	AB (autoaggress* OR “auto aggress*” OR automutilat* OR “auto mutilate*” OR cutt* OR overdos* OR (self N2 cut*) OR self-destruct* OR “self destruct*” OR selfharm* OR “self harm*” OR selfimmolat* OR “self immolate*” OR selfinflict* OR “self inflict*” OR selfinjur* OR “self injur*” OR selfmutilat* OR “self mutilate*” OR selfpoison* OR “self poison*” OR suicid*)
S22	AB suicid*
S21	TI suicid*
S20	S1 OR S2 OR S3 OR S4 OR S5 OR S6 OR S7 OR S8 OR S9 OR S10 OR S11 OR S12 OR S13 OR S14 OR S15 OR S16 OR S17 OR S18 OR S19
S19	AB (young N1 (people OR person))
S18	TI (young N1 (people OR person))
S17	AB schoolchild*
S16	TI schoolchild*
S15	AB pupil*
S14	TI pupil*
S13	AB youth*
S12	TI youth*
S11	DE "STUDENTS"
S10	AB student*
S9	TI student*
S8	DE "ADOLESCENT psychology"
S7	(DE "TEENAGERS") OR (DE "ADOLESCENCE")
S6	AB adolescen*
S5	TI adolescen*
S4	AB child*
S3	TI child*
S2	(DE "CHILD psychology") OR (DE "CHILD psychiatry")
S1	(DE "CHILDREN") OR (DE "CHILDREN -- Health")

Experts Consulted

Cathryn Rodway, Research Associate, University of Manchester (Responded, no additional papers identified)
Professor Kate Saunders, University of Oxford (Responded, no additional papers identified)
Professor Tamsin Ford, University of Exeter Medical School (Responded, no additional papers identified)
Professor Rory O’Connor, University of Glasgow (No response)

Excluded Papers with Reasons

Baytunca MB, Ata E, Ozbaran B, Kaya A, Kose S, Aktas EO, et al. Childhood sexual abuse and supportive factors. Pediatrics International. 2017; 59(1): 10-5.	Conference abstract
Baytunca MB, Ata E, Ozbaran B, Kaya A, Kose S, Aktas EO, et al. Childhood sexual abuse and supportive factors. Pediatrics International. 2017; 59(1): 10-5.	Self-harm/suicidal ideation not an outcome
Beck NI, Arif I, Paumier MF, Jacobsen KH. Adolescent injuries in Argentina, Bolivia, Chile, and Uruguay: Results from the 2012-2013 Global School-based Student Health Survey (GSHS). Injury. 2016; 47(12): 2642-9.	Self-harm/suicidal ideation not an outcome
Berg-Kelly K, Eriksson J. Adaptation of adopted foreign children at mid-adolescence as indicated by aspects of health and risk taking - A population study. European Child and Adolescent Psychiatry. 1997; 6(4): 199-206.	Truancy and suicide but does not report association
Bowman S, McGorry P. The student safety net: a new angle for early intervention. Early Intervention in Psychiatry. 2016; 10(4): 279-81.	Editorial
Carli V, Hoven CW, Wasserman C, Chiesa F, Guffanti G, Sarchiapone M, et al. A newly identified group of adolescents at "invisible" risk for psychopathology and suicidal behavior: Findings from the SEYLE study. World Psychiatry. 2014; 13(1): 78-86.	Doesn't separate truancy from other risky behaviours
Carlini-Marlatt B, Gazal-Carvalho C, Gouveia N, De Fatima Marinho Souza M. Drinking practices and other health-related behaviors among adolescents of Sao Paulo City, Brazil. Substance Use and Misuse. 2003; 38(7): 905-32.	Absenteeism and suicide attempt but does not report association
Cash T. Rural Alaska Mentoring Project (RAMP). International Journal on School Disaffection. 2011; 8(1): 35-7.	Description of intervention
Centers for Disease C, Prevention. Health risk behaviors among adolescents who do and do not attend school--United States, 1992. MMWR - Morbidity & Mortality Weekly Report. 1994; 43(8): 129-32.	Self-harm/suicidal ideation not an outcome
Champion JD. Context of sexual risk behaviour among abused ethnic minority adolescent women. International Nursing Review. 2011; 58(1): 61-7.	Not attending school and suicidal ideation but does not report association
Chang HY, Chung Y, Keyes KM, Jung SJ, Kim SS. Associations between the timing of childhood adversity and adulthood suicidal behavior: A nationally-representative cohort. Journal of Affective Disorders. 2015; 186: 198-202.	Outcomes in adulthood
Chasimpha S, McLean E, Chihana M, Kachiwanda L, Koole O, Tafatatha T, et al. Patterns and risk factors for deaths from external causes in rural Malawi over 10 years: a prospective population-based study. BMC Public Health. 2015; 15: 1036.	Attendance not an exposure
Choquet M, Darves-Bornoz J-M, Ledoux S, Manfredi R, Hassler C. Self-reported health and behavioral problems among adolescent victims of rape in France: Results of a cross-sectional survey. Child Abuse & Neglect. 1997; 21(9): 823-32.	Absenteeism and suicide attempt but does not report association
Clarke T, Baker P, Watts C, Henderson H, Evans T, Sherr L. Self-harm in younger people: Audit of prevalence and provision. Psychology, Health & Medicine. 2001; 6(4): 349-59.	Mentions truancy and less presentations with self-harm in school holidays but does not report association
Conrad N. Where do they turn? Social support systems of suicidal high school adolescents. Journal of psychosocial nursing and mental health services. 1991; 29(3): 14-20.	Abstract says 'significantly different in terms of school performance and attendance' but this is not discussed in the paper
D'Aulerio M, Carli V, Iosue M, Basilico F, Recchia L, Apter A, et al. Prevalence of risk behaviours among European young. Preliminary data from an italian sample we-stay project. European Psychiatry. 2013; 28: no pagination.	Abstract only
Daniel SS, Walsh AK, Goldston DB, Arnold EM, Reboussin BA, Wood FB. Suicidality, School Dropout, and Reading Problems among Adolescents. Journal of Learning Disabilities. 2006; 39(6-): 507-14.	Exposure is school dropout
Du Roscoat E, Legleye S, Guignard R, Husky M, Beck F. Risk factors for suicide attempts and hospitalizations in a sample of 39,542 French adolescents. Journal of Affective Disorders. 2016; 190: 517-21.	Exposure is school dropout

Eaton DK, Brener N, Kann LK. Associations of Health Risk Behaviors with School Absenteeism. Does Having Permission for the Absence Make a Difference? Journal of School Health. 2008; 78(4): 223-9.	Outcome and exposure reversed
Flisher AJ, Chalton DO. High-school dropouts in a working-class South African community: Selected characteristics and risk-taking behaviour. Journal of Adolescence. 1995; 18(1): 105-21.	Exposure is school dropout
Gulec G, Aksaray G. Assessment of sociodemographic, sociocultural and family traits in young suicide attempters. Yeni Symposium. 2006; 44(3): 141-50.	Not English
Hanna GL, Fischer DJ, Fluent TE. Separation anxiety disorder and school refusal in children and adolescents. Pediatrics in Review. 2006; 27(2): 56-63.	Not original research – educational paper
Harel-Fisch Y, Abdeen Z, Walsh SD, Radwan Q, Fogel-Grinvald H. Multiple risk behaviors and suicidal ideation and behavior among Israeli and Palestinian adolescents. Social Science and Medicine. 2012; 75(1): 98-108.	Truancy and suicide but does not report association
Hill RM, Oosterhoff B, Kaplow JB. Prospective identification of adolescent suicide ideation using classification tree analysis: Models for community-based screening. Journal of Consulting and Clinical Psychology. 2017; 85(7): 702-11.	No association reported
Hutchinson PL, Ferrell N, Broussard M, Brown L, Chrestman SK. Can school choice improve more than just academic achievement? An analysis of post-Katrina New Orleans. The Journal of school health. 2014; 84(4): 221-32.	Missed school and suicide plan but does not report association
Ibrahim N, Sherina MS, Phang CK, Mukhtar F, Awang H, Ang JK, et al. Prevalence and predictors of depression and suicidal ideation among adolescents attending government secondary schools in Malaysia. Medical Journal of Malaysia. 2017; 72(4): 221-7.	Poor attendance not an exposure
Katsumata Y, Matsumoto T, Kitani M, Akazawa M, Hirokawa S, Takeshima T. School problems and suicide in Japanese young people. Psychiatry and Clinical Neurosciences. 2010; 64(2): 214-5.	Letter
Knight A, Havard A, Shakeshaft A, Maple M, Snijder M, Shakeshaft B. The feasibility of embedding data collection into the routine service delivery of a multi-component program for high-risk young people. International Journal of Environmental Research and Public Health. 2017; 14(2): no pagination.	Attendance not an exposure
Krakowski MI, Czobor P. Psychosocial risk factors associated with suicide attempts and violence among psychiatric inpatients. Psychiatric Services. 2004; 55(12): 1414-9.	Outcomes in adulthood
Kwangu M, Mulenga D, Mazaba ML, Njunju EM, Siziya S. Adolescents attending school in the Philippines and suicidal ideation. In: Suicide: A global view on suicidal ideation among adolescents: 51-61. Nova Biomedical Books; US, 2017.	Book chapter
Kwangu M, Mulenga D, Mazaba ML, Njunju EM, Siziya S. Prevalence and factors for suicide ideation among adolescents attending school in Ghana. In: Suicide: A global view on suicidal ideation among adolescents: 21-31. Nova Biomedical Books; US, 2017.	Book chapter
Kwangu M, Njunju EM, Mulenga D, Mazaba ML, Siziya S. Oman and suicidal ideation among school-going adolescents. In: Suicide: A global view on suicidal ideation among adolescents: 43-50. Nova Biomedical Books; US, 2017.	Book chapter
Kweon Y, Kim AR, Cho HN. Suicidal behavior and related factors among youth students in South Korea. Journal of the American Academy of Child and Adolescent Psychiatry. 2017; 56 (10): S223-S4.	Conference abstract
Lacina, R. M., Staub-Ghielmini, S., Bircher, U., Bianchi, F., Schmeck, K., & Schmid, M. (2014). Survey of problematic behaviour of adolescents and their self-reported psychopathology. [Die erfassung von problemverhalten bei jugendlichen und ihre psychische belastung im selbsturteil.]. *Praxis der Kinderpsychologie und Kinderpsychiatrie, 63*(1), 36-62.	Not English
Lazzarini T, Rohrbaugh RM, Croda J, Goncalves C, Ko A, Benites W, et al. Adolescent suicide among the Guarani-Kaiowa in Dourados, Mato Grosso do Sul, Brazil. Annals of Global Health. 2015; 81(1): 114.	Conference abstract
Lenzen, C., & Brunner, R. (2013). Working in europe to stop truancy among youth (WE-STAY) project: Preventing truancy and promoting mental health of adolescents in different European countries. *European Child and Adolescent Psychiatry, 22*(3), 197-198. doi:http://dx.doi.org/10.1007/s00787-013-0387-9	Conference abstract
Lim F, Wong J, Loh A, Nyein N, Lei F, Chong PK, et al. Critical life stressors among adolescent suicide attempters in Singapore. Annals of the Academy of Medicine Singapore. 2014; 43(9 SUPPL. 1): S139.	Conference abstract

Lueck C, Kearl L, Lam CN, Claudius I. Do emergency pediatric psychiatric visits for danger to self or others correspond to times of school attendance? American Journal of Emergency Medicine. 2015; 33(5): 682-4.	Comparison of school holidays vs term time
Madianos MG, Gefou-Madianou D, Stefanis CN. Depressive symptoms and suicidal behavior among general population adolescents and young adults across Greece. European Psychiatry. 1993; 8(3): 139-46.	Enrolled in school vs not enrolled
Mazaba ML, Siziya S, Merrick J. Suicide: A global view on suicidal ideation among adolescents. In: Suicide: A global view on suicidal ideation among adolescents: xiii, 253. Nova Biomedical Books; US, 2017.	Book chapter
Mazaba ML, Siziya S, Mulenga D, Njunju EM, Kwangu M. Suicidal ideation among adolescents attending school in Kuwait. In: Suicide: A global view on suicidal ideation among adolescents: 141-51. Nova Biomedical Books; US, 2017.	Book chapter
Mazza JJ, Eggert LL. Activity Involvement among Suicidal and Nonsuicidal High-Risk and Typical Adolescents. Suicide and Life-Threatening Behavior. 2001; 31(3): 265-81.	Exposure is school dropout
McGibben L, Ballard CG, Handy S, Silveira WR. School attendance as a factor in deliberate self-poisoning by 12-15 year old adolescents. British Medical Journal. 1992; 304(6818): 28.	Comparison of school holidays vs term time
Peltzer, K., & Pengpid, S. (2012). Suicidal Ideation and Associated Factors among School-Going Adolescents in Thailand. *International Journal of Environmental Research and Public Health, 9*(2), 462-473. doi:10.3390/ijerph9020462	Reporting on same sample
Potthoff, S. J., Bearinger, L. H., Skay, C. L., Cassuto, N., Blum, R. W., & Resnick, M. D. (1998). Dimensions of risk behaviors among American Indian youth. *Archives of Pediatrics and Adolescent Medicine, 152*(2), 157-163.	No association reported
Pratt HD, Greydanus DE. Adolescent violence: concepts for a new millennium. Adolescent medicine (Philadelphia, Pa). 2000; 11(1): 103-25.	Literature review
Ribakoviene V, Puras D. [Relationships between social factors and suicidal attempts of adolescent girls]. Medicina (Kaunas, Lithuania). 2002; 38(4): 379-86.	Not English
Rivers I. Social exclusion, absenteeism and sexual minority youth. Support for Learning. 2000; 15(1): 13-8.	Outcomes in adulthood
Rojas Y, Stenberg SA. Early life circumstances and male suicide - A 30-year follow-up of a Stockholm cohort born in 1953. Social Science and Medicine. 2010; 70(3): 420-7.	Outcomes in adulthood
Shaikh MA. Prevalence, Correlates, And changes in tobacco use between 2006 and 2010 among 13-15 year moroccan school attending adolescents. Journal of the Pakistan Medical Association. 2014; 64(11): 1306-9.	Letter
Stein K, Fazel M. Depression in young people often goes undetected. The Practitioner. 2015; 259(1782): 17-3.	Not original research – educational paper
Suss AL, Tinkelman BK, Freeman K, Friedman SB. School attendance, health-risk behaviors, and self-esteem in adolescents applying for working papers. Bulletin of the New York Academy of Medicine. 1996; 73(2): 255-66.	Exposure is school dropout
Tammariello AE, Gallahue NK, Ellard KA, Woldesemait N, Jacobsen KH. Parental involvement and mental health among Thai adolescents. Advances in School Mental Health Promotion. 2012; 5(4): 236-45.	Skipping school and suicidality but does not report association

Data Extraction

Reference:

Self-harm variable
Type and duration:

Binary/other:

Method of ascertainment:

Attendance/exclusion variable
Type and duration:

Binary/other:

Method of ascertainment:

Covariates:

N subjects (or cases/controls):

Characteristics of cohort:

Country:

Age (mean/median and range):

Study design:

Time period of data collection:

Odds ratio(s) or other effect estimate(s):

Confidence interval(s):

P Value(s):

Notes about results:

Quality assessment details: (see quality assessment tools).

Exposure and outcome variables

	Exposure	Outcome
Almansour & Siziya 2017	Truancy	Did you ever seriously consider attempting suicide?
Asante et al 2017	During the past 30 days, how many days did you miss classes or school without permission? (3 or more times vs less than 3 times)	Three main outcome measures were extracted from the data, namely, suicidal ideation, suicidal plan and suicidal attempts. In this study, each of these three outcome variables was measured with a single self-report item or question. For example, the item, "during the past 12 months, did you ever seriously consider attempting suicide?" was used to measure suicidal ideation while suicidal plan was measured with the question, "during the past 12 months, did you make a plan about how you would attempt suicide?". The responses were categorized as "yes" (1) or "no" (0). Suicidal attempt was measured with the question "during the past 12 months, how many times did you actually attempt suicide?" The responses for this questions were "0", "1", "2 or 3", "4 or 5", and "6 or more times". The responses were recoded as no attempt (0) and one or more attempts (1) for analysis.
Bailey et al 2014	Contempt of Court for Truancy was defined as a truant youth who missed school after appearing before a judge for truancy, or a truant youth who did not participate in court-ordered community service or programme as ordered by the Judge or Justice of the Peace. Severe truancy, for this study, was used interchangeably with contempt of court for truancy.	All injury-related deaths among children <18 years of age for which the incident occurred in Dallas County. Included in this study were all youth who died due to injury (homicide, suicide or unintentional injury) during the study period.
Bjarnason & Thorlindsson 1994	How often do you play truant from school? Never, <monthly, monthly, weekly, daily	Dichotomous response to the question "have you ever tried to kill yourself?"
Borowsky et al 1999	'Skipped school in last month': yes or no)	A history of ever having attempted suicide was assessed with the question: "Have you ever tried to kill yourself?"
Borowsky et al 2001	This school year, how often skipped school	The Time 2 outcome variable was assessed with the question: "During the past 12 months, did you actually attempt suicide?"
Brunner et al 2013	Truancy From Wasserman et al 2010 '2-week measure, when missed 3 or more days of school or class without permission'	Lifetime direct self-injurious behaviour (D-SIB) defined as the intentional self-inflicted damage of the surface of an individual's body by self-cutting, -burning, -hitting, -biting, and skin damage by other methods. (a) Have you ever intentionally cut your wrist, arms, or other area(s) of your body, or stuck sharp objects into your skin such as needles, pins, staples (NOT INCLUDING tattoos, ear piercing, needles used for drugs, or body piercing)? (b) Have you ever intentionally burned yourself with a cigarette, lighter, or match? (c) Have you ever intentionally carved words, pictures, designs, or other markings into your skin, or scratched yourself to the extent that scarring or bleeding occurred? (d) Have you ever intentionally prevented wounds from healing, or bit yourself to the extent that it broke skin? (e) Have you ever intentionally banged your head or punched yourself thereby causing a bruise? (f) Have you ever intentionally hurt yourself in any of the above-mentioned ways so that it led to hospitalization or injury severe enough to require medical treatment? Occasional D-SIB was defined as 1–4 reported lifetime acts of D-SIB; repetitive D-SIB was defined as ≥5 previous events of D-SIB acts during lifetime. The cut-off of ≥5 has been chosen according to the diagnostic criteria of frequency in the new proposed

		diagnostic entity of NSSI according to DSM-5 (American Psychiatric Association, 2013)
Chen et al 2005	Number of days absent from school in the past 30 days	“During the past 12 months, did you seriously consider ending your life?” “During the past 12 months, did you make a plan about how you would end your life?”; “During the past 12 months, how many times did you actually attempt suicide?”
Cheng et al 2009	‘During the past 30 days, on how many days did you miss classes or school without permission? 0, 1-5, 6 or more’	‘During the past 12 months, did you ever seriously consider attempting suicide? During the past 12 months, how many times did you actually attempt suicide?’ Dichotomised.
Choquet & Menke 1990	‘Frequent school related problems in past 12 months: Absent from class’	Suicidal thoughts over the past 1 year – ‘never’, ‘rarely’, ‘fairly often’, ‘very often’
Cwik et al 2015	Days missed school because feared unsafe (past 30) (0,1,2-3,4-5,6+)	Multiple suicide attempts in past 90 days (compared to single suicide attempt)
Davaasambuu et al 2017	“During the past 30 days, on how many times did you miss classes or school without permission?” - then dichotomised to 0 or 1+ times	“During the past 12 months, have you made a plan about how you would attempt suicide?” “During the past 12 months, how many times have you actually attempted suicide?” – then dichotomised to 0 or 1+ times
De Man et al 1993	Absenteeism in terms of periods absent	Suicidal ideation assessed with the Scale for Suicide Ideation (de Man et al 1993)
Donath et al 2014	“I have so far never been truant a whole day” All students who did not check the item received a “positive” truancy score.	“Have you ever seriously tried to commit suicide?”
Epstein & Spirito 2009	Skipped school because unsafe (yes/no) - but no timing in table. In text described as 'felt unsafe at school or on their way to or from school on one or more of the past 30 days'	Past 12 months – “did you ever seriously consider attempting suicide?” “Did you make a plan about how you would attempt suicide?”. “How many times did you attempt suicide” (0 vs 1 or more)
Evren et al 2014	Absenteeism (none, 1-14 days, more than 15 days)<br>Truancy before age 13	Self-harm behaviour (SHB) within the past year. Self-harm was defined as “deliberate self-injury to body tissue without the intent to die.” The SHB question was “Do you harm yourself intentionally? (never/at least once)” . The question also included the most commonly used methods of SHB in parentheses (cutting, burning, hitting oneself, inserting sharp objects into body orifices and pulling out body hair)
Fergusson et al 2003	School truancy age 11-15	Suicidal thoughts since the previous assessment at age 15, 16, 18 and 21. (Previous interviews age 14)
Kandel et al 1991	Never cuts class	The suicidal ideation scale was developed from 4 items. The first item, taken from the SCL-90 Depression Scale (Derogatis, 1977), asks respondents, "How much have you been bothered by thoughts of ending life in the last twelve months?" The second, from the Beck Depression Inventory (Beck et al., 1961), is "Thoughts of killing myself during the past few weeks", with response alternatives ranging from “Don't have any thoughts”, “Have thoughts but would not carry them out”, “Would like to kill myself”, to “Would kill myself if I had the chance.” The third and fourth items are from the Zung Index of Potential Suicide (Zung, 1974), and asks how much of the time the following statements apply: "I've said to someone that I wanted to kill myself" and "I've been thinking of ways to kill myself." A scale ranging from 1 to 3 was created by assigning a score of 1 to respondents

		who responded negatively to each item, 2 if they indicated experiencing suicidal thoughts or talked about suicide at most a little of the time (SCL-90 or Zung items) or thought of suicide but would not kill themselves (Beck item), and 3 if they reported stronger suicidal feelings, stating at least that they would like to kill themselves (Beck item) or that they have thoughts of killing themself, spoke of killing themself (Zung items), or are bothered by suicidal thoughts (SCL-90 item) at least some of the time (Cronbach's alpha .82).
Larsson and Sund 2008	Frequency of truancy within the last year (None to more than once a month) measured at T1 assessed on a 1-4 scale (good to bad)	Overdose/Deliberate Self Harm (DSH) without suicidal intent. Suicide attempt. Lifetime at T1 and last 12 months at T2 one year later - latter used as outcome
Lau et al 2010	Absence from school since the earthquake occurred while the school was not closed	Suicidal ideation: "Whether you have thought about committing suicide since the May 12 earthquake?" (1 month)
Lewinsohn et al 1993	Days missed in school in past 6 weeks	Past suicide attempt (lifetime). "Have you ever tried to kill yourself or done anything that could have killed you?"
Lewinsohn et al 1994	Missed school days in the last 6 weeks	Suicide attempt between first and second assessments (one year apart). "Have you ever tried to kill yourself or done anything that could have killed you?"
Lyon et al 2000	Truancy	Presenting to paediatric hospital following suicide attempt (controls = walk-in patients who used the adolescent primary care clinic, with no history of suicide attempt).
Noble et al 2011	'During the past 30 days, how many times did you NOT go to school because you felt you would be unsafe at school or on your way to or from school?'	Participants asked whether they had ever hurt themselves to deal with stress or other problems (non suicidal self-injury). Suicide attempts excluded. Controls had never injured themselves.
Pages et al 2004	School absenteeism in last 12 months (never, sometimes, often)	Suicide attempts with and without hospitalisation. (Items concerning suicide attempts ("During lifetime, how many times have you attempted suicide: no/once/twice and more") and hospitalization ("If you made a suicide attempt, were you hospitalized for this reason: no/once/twice and more").)
Pillai et al 2009	Number of days absent from school in the past 3 months. 0 days, 1-3 days, 4-6 days, 7 days or more	Three questions to elicit suicidal behaviour—whether seriously considered ending one's life; made a plan about ending one's life; or attempting suicide. The reference period for all three questions was the 3 months before the interview, and a positive response to at least one of the questions was considered as suicidal behaviour. Used the term suicidal behaviour to include suicidal thoughts, plans and attempts.
Peltzer & Pengpid 2017	Truancy: "During the past 30 days, on how many days did you miss classes or school without permission?" (Recoded 0 = 0 times, 1 = 1 or more times)	'During the past 12 months, did you ever seriously consider attempting suicide?'
Randall et al (2014)	"During past 30 days, on how many days did you miss classes or school without permission" (1-2 vs 3 or more days)	"During the past 12 months, did you ever seriously consider attempting suicide?'' and ''During the past 12 months, did you make a plan about how you would attempt suicide?''. ''During the past 12 months, how many times did you actually attempt suicide?''. (0, 1 or more than one)

Sharma et al 2015	"During the past 30 days how many days did you miss class without giving notice to the school?" (0-2 days or 3+ days)	Suicidal ideation in past 12 months<br>Suicide attempt in past 12 months "In the past 12 months, have you tried to end your life?"
Taliaferro & Muehlenkamp 2014	On a 5-point scale, during the last 30 days, how many days did not go to school because felt would be unsafe at school or on the way to or from school (dichotomised to 0 times/1 or more times)	"Have you ever thought about killing yourself?" and "Have you ever tried to kill yourself?". Response items for both items were: "no", "yes, during the last year," or "yes, more than a year ago." To ensure data had relevance to currently suicidal youth, positive responses to "during the last year" were used to drive group classifications. Students were classified into three groups: suicidal ideation only (thought about killing oneself during the last year and never attempted suicide), suicide attempt (attempted suicide during the last year, with any reported suicidal ideation), and no suicidality (never thought about or attempted to kill oneself ever). Youth who only endorsed "more than a year ago" on both items were excluded from the analysis to avoid confounding the classification of youth into current suicide ideation and attempter groups.
Wilson et al 2012	"During the past 30 days, on how many days did you miss classes or school without permission?". Response items were "0", "1–2", "3–5", "6–9" and "10 or more days". Students were considered truant if they missed more than 3 days of school within the reference period using a prior threshold by Wilson et al. [17].	"During the past 12 months, did you ever seriously consider attempting suicide?" "During the past 12 months, did you make a plan about how you would attempt suicide?"
Xin et al 2017	Truancy	'In the past 12 months, (a) Have you ever intentionally cut or scalded yourself? (b) Have you ever intentionally bit, scratched, or hit yourself?' Direct Self Injurious Behaviour was recorded if a participant answered "yes" to either item.

Full List of Covariates

Reference	Covariates
Almansour et al 2017	Age, gender, food security, anxiety, loneliness, close friends, bullied, attacked, in a fight, ever used drugs, ever used marijuana, parental understanding
Asante et al 2017	Age, anxiety, loneliness, bullied, attacked, in a fight, food insecurity, close friends, smoking, substance use, alcohol misuse, parental homework checking, parental understanding, parental knowledge of activity, parental intrusion of privacy
Bailey et al 2014	None
Bjarnason et al 1994	School factors, leisure, peers and friends, consumption, parents, suicide suggestion
Borowsky et al 1999	None
Borowsky et al 2001	Age, Family structure, Welfare status
Brunner et al 2013	Gender, country, age, student perceived himself/herself as a religious person, parental unemployment, student does not live with biological parent or relative, parents do not understand student's problems, parents do not pay attention to student, loneliness/social relationship problems, peer victimization, depression, anxiety, suicidality, sensation-seeking and delinquent behaviors, tobacco use, alcohol/drug consumption
Chen et al 2005	Gender, education level, smoking, drinking alcohol, taking drugs (Ganja), living with parents, level of violence (physical fight), injury in physical fight, carrying weapons to school, number of days felt unsafe to go to school in past 30 days, feeling sad or loss of hope in the past 14 days, and drunk driving or riding in a car driven by a drunk driver. (Only urban students in multivariate analysis)
Cheng et al 2009	City, gender, age, peers kind and helpful, parents check homework, parents understand problems and worries, parents know what you do
Choquet & Menke 1990	None
Cwik et al 2015	None
Davaasambuu et al 2017	Gender, feeling lonely, feeling worried, smoking cigarettes, drinking alcohol, physical fight, having friends, peers helpful, bullied, residential location (rural vs urban)
De Man et al 1993	Demographic and family characteristics, school achievement, self-esteem, locus of control, depression, life stress, parental control, social support, feelings of alienation (unclear which were included in multivariate model)
Donath et al 2014	Parenting styles: authoritative, permissive, authoritarian, rejecting-neglecting. Age, sex, migration background, welfare status, binge drinking, smoking, ADHD, parental separation, non-profit volunteer activities, school grades, school anxiety, mental wellbeing, self-esteem, social integration in school
Epstein et al 2009	Carried weapon past 30 days, threatened at school 12 months, property stolen at school 12 months, fought past 12 months, hit by girl/boyfriend
Evren et al 2014	None – exposures not included in multivariate model
Fergusson et al 2003	Not clear what is controlled for
Kandel et al 1991	None
Larsson and Sund 2008	None - Truancy not included in multivariate model
Lau et al 2010	Gender, age, grade, previous adversities (death of relatives, bullying, serious accident, severe mental illness, corporal punishment), direct impact of earthquake (temporary departure from Chengdu, number of nights stayed indoors, number of hours losing contact with family members, parents were in Chengdu 1st week after earthquake, parents went to affected area after earthquake), responses to the earthquake (support from parents/teachers/peers), impact from media, worry about future disasters, children's revised impact of events scale score (CRIES)
Lewinsohn et al 1993	Gender<br>And then depression
Lewinsohn et al 1994	Gender
Lyon et al 2000	Kept in model: insomnia, threat of separation, alcohol/drug abuse, neglect, academic problems, suicidal ideation, age, threatens others, separated from parental figure >2 weeks
Noble et al 2011	Trust students, trust teachers, trust administration, trust school counsellor, carried weapon to school, threatened at school, bullied at school, school fights
Pages et al 2004	Age<br>In logistic regression model: Running away, Kandel score >17, tobacco 10+/day, Rosenberg score <26, repeated consumption of illicit drugs other than cannabis, consumption of cannabis, physical fighting
Peltzer et al 2017	Gender, age, hunger, country income, psychosocial distress (no close friends, loneliness, bullied, attacked, in physical fight), parental/guardian support, current tobacco, ever got drunk, physical activity <60 min on at least 5 days/week , sitting >3 hours/day, BMI overweight or obesity (fewer covariates included in models for each country separately)
Pillai et al 2009	Gender, age, area of residence (urban/rural). Currently studying, living with parents vs others, easy to talk to parents, able to make own decisions, sexual relations (unmarried).
Randall et al 2014	Age, gender, anxiety, loneliness, bullied, attacked, in a fight, food insecurity, alcohol misuse, substance use, tobacco use, parental support, close friends
Sharma et al 2015	In fight, insulted, attacked, parental understanding, spending with parents, parental homework checking

Taliaferro et al 2014	White vs non-white, grade 9 vs grade 12, free or reduced price lunch, live with both biological parents vs all other parent options, victim of social-verbal bullying, perpetrator of social-verbal bullying, family substance abuse, witness to family violence, physical abuse, sexual abuse, victim of dating violence, mental health problem, physical health problem, cigarette smoking, alcohol use, binge drinking, marijuana use, misuse of prescription drugs or use of illegal drugs, ran away from home, self-injury, perpetrator of violence, victim of school violence, perceived overweight or engage in maladaptive dieting behaviour, same-sex sexual experience, depressive symptoms, hopelessness, stress or anxiety, distractibility/impulsivity
Wilson et al 2012	None – truancy not included in multivariate analyses
Xin et al 2017	Age, paternal education level

Reasons for excluding from meta-analysis

Reference	Reason
Bailey et al 2014	Cohort study (all included were cross sectional) with outcome of completed suicides (so not comparable with other cohort studies)
Bjarnason et al 1994	No odds ratios reported
Borowsky et al 1999	No odds ratios reported
Borowsky et al 2001	Ethnicities reported separately, no result for whole cohort so not comparable
Chen et al 2005	Exposure is a continuous variable (all included were binary)
Cheng et al 2009	Categorical exposure variable (all included were binary)
Choquet & Menke 1990	No adjusted odds ratios reported
Cwik et al 2015	No adjusted odds ratios reported
De Man et al 1993	No odds ratios reported
Epstein et al 2009	Not included in suicidal ideation meta-analysis as no odds ratios reported
Evren et al 2014	No adjusted odds ratios reported
Fergusson et al 2003	Cohort study so not comparable (all included were cross sectional)
Kandel et al 1991	No adjusted odds ratios reported
Larsson and Sund 2008	Cohort study (all included were cross sectional), no adjusted odds ratios reported
Lau et al 2010	Young people recently exposed to an earthquake, not comparable population
Lewinsohn et al 1993	Unclear whether exposure variable is binary or continuous
Lewinsohn et al 1994	Cohort study (all included were cross sectional), no adjusted odds ratios reported
Lyon et al 2000	Case control study (all included were cross sectional) in clinical sample (so not comparable with other case control study)
Noble et al 2011	Case control study (all included were cross sectional) in community sample (so not comparable with other case control study)
Pages et al 2004	Adjusted analysis reported for male subgroup only
Pillai et al 2009	Categorical exposure variable (all included were binary)
Randall et al 2014	Categorical outcome variable (multinomial logistic regression)
Taliaferro et al 2014	Male and female subgroups reported separately
Wilson et al 2012	No adjusted odds ratios reported

Funnel plots for studies included in meta-analyses

Poor school attendance and self-harm

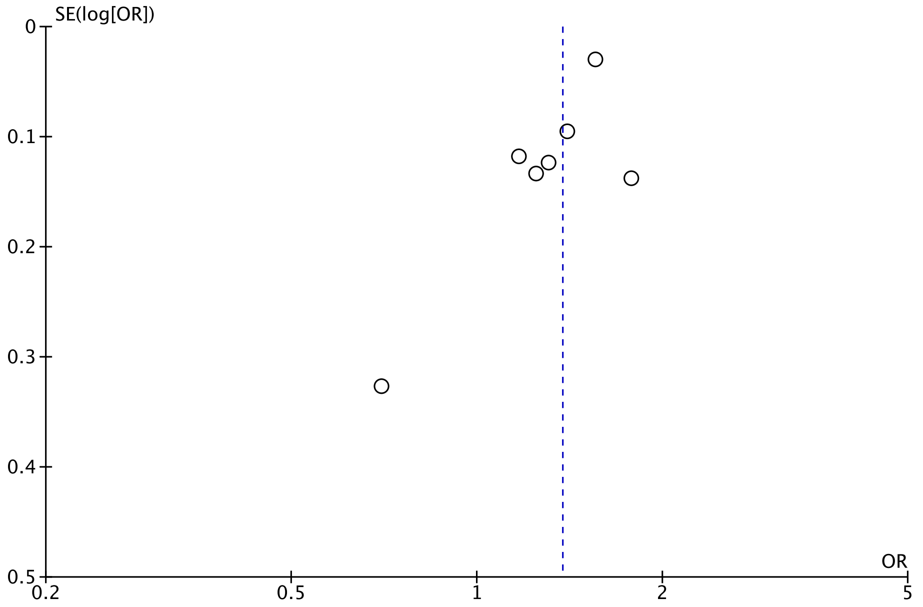

Egger's test for small-study effects:
Test of H0: no small-study effects P = 0.568

Poor school attendance and suicidal ideation

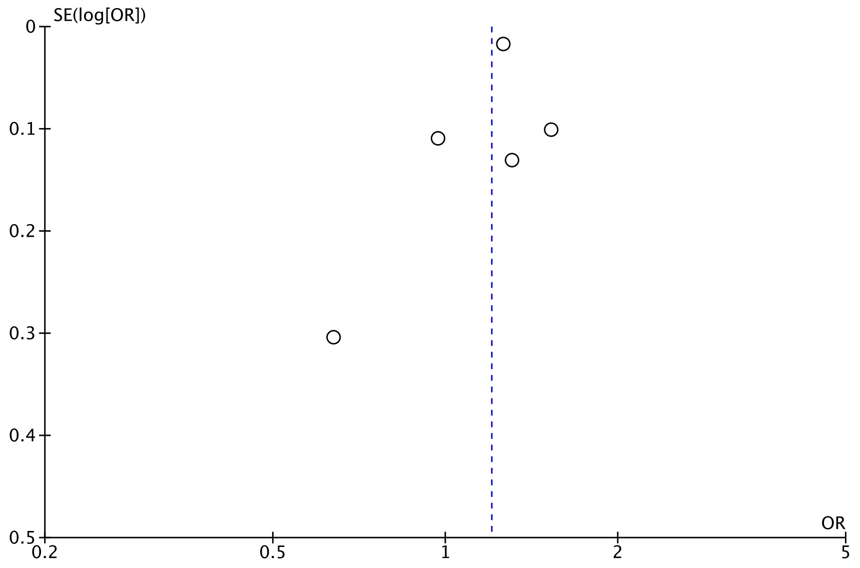

Egger's test for small-study effects:
Test of H0: no small-study effects P = 0.057

Note: results should be interpreted with caution given the small number of studies in each meta-analysis.

Quality assessment tools: Adapted Newcastle-Ottawa Quality Assessment Scale

<u>Cross-Sectional Studies</u>

Note: A study can be awarded a maximum of one point for each numbered item with the Selection and Exposure categories. A maximum of two points can be given for Comparability.

Selection (maximum 4 stars*)

1. <u>Representativeness of the sample</u>
    a. Truly representative of the average in the target population (random sample or whole population) *
    b. Somewhat representative of the average in the target population (purposive sampling of representative schools or evidence that the sample is representative of the source population) *
    c. Selected group of users
    d. No description of the sampling strategy
2. <u>Sample size</u>
    a. Justified and satisfactory *
    b. Adequately powered to detect a difference (at least 10 events per variable in multivariate analyses)*
    c. Not justified
3. <u>Non-respondents</u>
    a. Comparability between respondents and non-respondents characteristics is established, and the response rate is satisfactory (>60%)*
    b. The response rate is unsatisfactory, or the comparability between respondents and non-respondents is unsatisfactory
    c. No description of the response rate or the characteristics of the responders and non-responders
4. <u>Ascertainment of the exposure (absence or exclusion)</u>
    a. School administrative records*
    b. Reported by school staff*
    c. Other collateral or self-report
    d. No description

Comparability (maximum 2 stars*)

5. <u>The subjects in different outcome groups are comparable, based on the study design or analysis. Confounding factors are controlled</u>
    a. Study controls for age and gender (or analysis separated by gender)*
    b. Study controls for any additional factor *

Outcome (maximum 2 stars*)

6. <u>Assessment of the outcome (self-harm or suicidal ideation)</u>
    a. Structured interview or written self-report*
    b. Clinical record*
    c. Reported by school or collateral
    d. No description

7. <u>Statistical test</u>

    a. The statistical test used to analyse the data is clearly described and appropriate, and the measurement of the association is presented as either an OR, CI and P value or a beta coefficient, SE and P value*
    b. The statistical test is not appropriate, not described or incomplete

Additional parameter not in NOS:

8. Clear variables (Maximum 2)
    a. Both variables clearly defined including time period for measurement (2)
    b. One or other variable clearly defined including time period for measurement (1)
    c. Neither variable clearly defined including time period for measurement (0)

***

Cohort Studies

Note: A study can be awarded a maximum of one point for each numbered item with the Selection and Exposure categories. A maximum of two points can be given for Comparability.

Selection (maximum 4 stars*)

1. Representativeness of the cohort
    a. Truly representative of the average in the target population (random sample or whole population)*
    b. Somewhat representative of the average in the target population (purposive sampling of representative schools or evidence that the sample is representative of the source population)*
    c. Selected group of users
    d. No description of the derivation of the cohort
2. Selection of the non-exposed cohort
    a. Drawn from the same community as the exposed cohort *
    b. Drawn from a different source
    c. No description of the derivation of the non-exposed cohort
3. Ascertainment of exposure (absence or exclusion)
    a. School administrative records*
    b. Reported by school staff*
    c. Other collateral or self-report
    d. No description
4. Demonstration that outcome of interest was accounted for or not present at start of study
    a. Yes *
    b. No

Comparability (maximum 2 stars*)

5. Comparability of cohorts on the basis of the design or analysis
    a. Study controls for age and gender (or analysis separated by gender) *
    b. Study controls for any additional factor *

Outcome (maximum 3 stars*)

6. Ascertainment of outcome (self-harm or suicidal ideation)
    a. Structured interview or written self-report*
    b. Clinical record*
    c. Reported by school or collateral
    d. No description

7. Was follow-up long enough for outcomes to occur?
    a. Yes (>1 year) *
    b. No
8. Adequacy of follow-up of cohorts
    a. Complete follow-up – all subjects accounted for *
    b. Subjects lost to follow-up unlikely to introduce bias – small number lost (<20%) or attrition described and accounted for in analysis *
    c. Follow up rate not adequate and no description of those lost
    d. No statement

Note: A study can be awarded a maximum of one point for each numbered item for sample size and statistical test. A maximum of two points can be given for clear variables.

Additional parameters not in NOS:

Not in NOS, taken from cross sectional NOS:

9. Sample size (Maximum 1)
    a. Justified and satisfactory *
    b. Adequately powered to detect a difference (at least 10 events per variable in multivariate analyses)*
    c. Not justified

10. Statistical test (Maximum 1)
    a. The statistical test used to analyse the data is clearly described and appropriate, and the measurement of the association is presented as either an OR, CI and P value or a beta coefficient, SE and P value*
    b. The statistical test is not appropriate, not described or incomplete

Further additional parameter:

11. Clear variables (Maximum 2)
    a. Both variables clearly defined including time period for measurement (2)
    b. One or other variable clearly defined including time period for measurement (1)
    c. Neither variable clearly defined including time period for measurement (0)

***

Case-Control Studies

Note: A study can be awarded a maximum of one point for each numbered item with the Selection and Exposure categories. A maximum of two points can be given for Comparability.

Selection (maximum 4 stars*)

1. Is the case definition adequate? (self-harm or suicidal ideation)
    d. Structured interview or written self-report*
    e. Clinical record*
    f. Reported by school or collateral
    g. No description
2. Representativeness of the cases
    a. Consecutive or obviously representative series of cases *
    b. Potential for selection biases or not stated
3. Selection of controls
    a. Community controls from source population*

   b. Clinical controls if clinical source population*
   c. Not extracted from same source population
   d. No description
4. Definition of controls
   a. No history of outcome (NB if cases have new (not necessarily first) occurrence of outcome, controls with previous occurrences should not be excluded) *
   b. No description of source

Comparability (maximum 2 stars*)

5. Comparability of cases and controls on the basis of the design or analysis
   a. Study controls for age and gender (or analysis separated by gender) *
   b. Study controls for any additional factor *

Exposure (maximum 3 stars)

6. Ascertainment of exposure (absence or exclusion)
   a. School administrative records*
   b. Reported by school staff*
   c. Other collateral or self-report
   d. No description
7. Same method of ascertainment for cases and controls
   a. Yes *
   b. No
8. Non-response rate
   a. Same rate for both groups *
   b. Non-response or missing values should be <20% and accounted for in analysis*
   c. Non respondents described
   d. Rate different and no designation

Note: A study can be awarded a maximum of one point for each numbered item for sample size and statistical test. A maximum of two points can be given for clear variables.

Additional parameters: Not in NOS, taken from cross sectional NOS:

9. Sample size (Maximum 1 star)
   a. Justified and satisfactory *
   b. Adequately powered to detect a difference (at least 10 events per variable in multivariate analyses)*
   c. Not justified

10. Statistical test (Maximum 1 star)
    a. The statistical test used to analyse the data is clearly described and appropriate, and the measurement of the association is presented as either an OR, CI and P value or a beta coefficient, SE and P value*
    b. The statistical test is not appropriate, not described or incomplete

Further additional parameter:

11. Clear variables (Maximum 2 stars)
    a. Both variables clearly defined including time period for measurement (2)
    b. One or other variable clearly defined including time period for measurement (1)
    c. Neither variable clearly defined including time period for measurement (0)

Results of quality assessment

Quality Assessment: Poor attendance and suicidal ideation

Cohort studies

Reference	Representativeness of exposed cohort (0-1)	Selection of non-exposed cohort (0-1)	Ascertainment of exposure (0-1)	Outcome not present at start (0-1)	Comparability - adjustment for confounders (0-2)	Ascertainment of self-harm (0-1)	Follow-up long enough (0-1)	Adequacy of follow up (0-1)	Sample size (0-1)	Statistical reporting (0-1)	Clear variables? (0-2)	Total (Max 13)
Fergusson 2003	1<br><br>Unselected birth cohort	1<br><br>One cohort containing exposed and non-exposed	0<br><br>Self and parental reports	1<br><br>Since previous assessment	0<br><br>Not clear what is controlled for or if truancy/suspension included or non-significant in final model	1<br><br>Self-report	1<br><br>Outcomes measured between age 15 and 21, followed up since age 1	1<br><br>84% of the original cohort had data on suicidal behaviour. Sample bias statistically managed	1<br><br>Analysis performed which showed sample bias minimal	1<br><br>B coefficient, SE and P value	1<br><br>Suicidal behaviour unclear over what time period reported in results, suspensions before age 15	**9**

Cross sectional studies

Reference	Representativeness of sample (0-1)	Sample size (0-1)	Non-respondents (0-1)	Ascertainment of exposure (0-1)	Comparability - adjustment for confounders (0-2)	Ascertainment of self-harm (0-1)	Statistical reporting (0-1)	Clear variables? (0-1)	Total (Max 10)
Almansour 2017	0<br><br>Not random	1<br><br>>10 Events Per Variable in multivariate analysis (EPV)	1<br><br>97% response rate	0<br><br>Self-reported	2<br><br>Age, gender and others	1<br><br>Self-reported	0<br><br>OR, CI but not P value	1<br><br>Suicidal ideation past 12 months but truancy not clear	**6**
Asante 2017	**1**<br><br>Random sample of classes	**1**<br><br>>10 EPV	**0**<br><br>Response rate not stated	**0**<br><br>Self-reported	**2**<br><br>Age, gender and others	**1**<br><br>Self-reported	1<br><br>OR and CI and value	2<br><br>12 months suicidal behaviour and 30 day poor attendance	**8**
Chen 2005	1<br><br>Random sampling of schools and classes in several districts	1<br><br>>10 EPV (only urban included in multivariate model) Roughly 14 variables in analysis and roughly 150 events in urban group (states that	0<br><br>Dos not report how missing data was handled	0<br><br>Self-reported	2<br><br>Gender and grade for age, and others (only urban in multivariate)	1<br><br>Self-reported	0<br><br>OR, CI and P value. Not present for rural students where it was not significant	1<br><br>12-month suicidal ideation. Number of days absent not clear how used in analysis	**6**

		rates of events were similar between urban and rural groups)							
Cheng 2009	1<br><br>Random sample of schools and classes	1<br><br>>10 EPV	1<br><br>98.28% response rate	0<br><br>Self-reported	2<br><br>Age, gender and others	1<br><br>Self-reported	1<br><br>OR, CI and P value	2<br><br>Both clear	**9**
Choquet 1990	1<br><br>Random sample from region, 93% participation	1<br><br>Univariate	1<br><br>99% response rate (how is this different from the 93%?)	0<br><br>Self-administered questionnaire	0<br><br>None	1<br><br>Self-administered questionnaire	0<br><br>X2 and P values	0<br><br>Suicidal, never, rarely, often (not clear how define this), absent from class no definition	**4**
Davaasambu 2017	1<br><br>Randomised cluster sample from WHO survey	1<br><br>>10 EPV (roughly 24 variables - not clear re event rate for urban and rural separately but likely more than 250)	0<br><br>50 individuals excluded due to missing data - not checked for characteristics	0<br><br>Survey	2<br><br>Many including gender and age	1<br><br>Survey	1<br><br>OR, CI and P value	2<br><br>Clear variables	**8**
De Man 1993	1<br><br>Random cluster sampling, participation >98%	1<br><br>States 'sufficiently large sample size' showed by transformed data performing no better than original	1<br><br>98% participation rate	1<br><br>From school	0<br><br>Not clear what variables were in the multivariate model and some included when shouldn't be	1<br><br>Questionnaire	0<br><br>Correlation	0<br><br>Time periods not clear for either variable	**5**
Epstein 2009	1<br><br>Stratified sampling of schools then randomly within schools, nationally representative sample	1<br><br>N = 13917, events not mentioned	0<br><br>School response rate 78%, student 86% (overall response rate 67%) Doesn't say if non respondents differed	0<br><br>Questionnaire	1<br><br>Not age and gender but others included	1<br><br>Questionnaire	1<br><br>OR, CI and P value for suicide attempts	1<br><br>Suicide attempts in past year but skipping school variable not clear	**6**
Kandel 1991	1<br><br>Random sample from one school	1<br><br>Univariate	0<br><br>Not reported	0<br><br>Questionnaire	0<br><br>None	1<br><br>Questionnaire	0<br><br>% and P value	1<br><br>Complex scale for suicidality. Cutting class not clear how measured	**4**
Lau 2010	1<br><br>Two schools, 100% response rate	1<br><br>>10 EPV (29 variables)	1<br><br>100% response	0<br><br>Questionnaire	2<br><br>Gender, age and others	1<br><br>Questionnaire	1<br><br>OR, CI and P value	2<br><br>Both are clear – since the earthquake	**9**
Peltzer 2017	1	1	0	0	2	1	1	2	**8**

	Random cluster sampling,	>10 EPV for most groups - not for 2-3 countries (20 variables, 15 when done per country)	response rate between 82 and 96% in different countries Listwise deletion of cases with missing values	Questionnaire	Gender, age and others	Questionnaire	OR, CI and P value	Both clear, suicidal thoughts in 12 months, truancy in 30 days	
Randall 2017	1<br><br>Randomly selected classes	1<br><br>>10 EPV	1<br><br>90% response rate	0<br><br>Questionnaire	2<br><br>Gender, age and others	1<br><br>Questionnaire	1<br><br>RR, CI, P value	2<br><br>Ideation/plans/attempts over 12 months, miss school over 30 days	**9**
Sharma 2015	1<br><br>Randomly selected grades, schools and areas from 2 districts	1<br><br>Justified and calculation provided (also >10 EPV - 8 variables in model 1)	0<br><br>Some students missing on the day and others did not complete questions correctly, not accounted for	0<br><br>Questionnaire	2<br><br>Gender but not age (due to non-significance) included in regression model with absenteeism Yes others	1<br><br>Questionnaire	1<br><br>OR, CI and P value	2<br><br>Suicidal ideation and attempt in past 12 months. Missed school in past 30 days, clear parameters	**8**
Taliaferro 2014	1<br><br>88% of Minnesota districts, 60-80% of students – from Minnesota student survey	1<br><br>>10 EPV	0<br><br>Missing data not mentioned	0<br><br>Self-reported survey	2<br><br>Age and others included, genders analysed separately	1<br><br>Self-reported survey	1<br><br>OR, CI and P value	2<br><br>Both clear, lifetime suicidal thoughts/attempts. Missed school in last 30 days	**8**
Wilson 2012	1<br><br>WHO Global Student Health Survey but sampling method not mentioned	1<br><br>Univariate	0<br><br>Response rate 82%, characteristics not reported	0<br><br>Self-reported survey	2<br><br>Not included in multivariate model due to insignificance in univariate but model contains age and sex	1<br><br>Self-reported survey	0<br><br>Percentages and significance test – not clear what comparisons made	2<br><br>Clear definitions and time periods	**7**

Quality Assessment: Poor attendance and self-harm

Cohort studies

Reference	Representativeness of exposed cohort (0-1)	Selection of non-exposed cohort (0-1)	Ascertainment of exposure (0-1)	Outcome not present at start (0-1)	Comparability - adjustment for confounders (0-2)	Ascertainment of self-harm (0-1)	Follow-up long enough (0-1)	Adequacy of follow up (0-1)	Sample size (0-1)	Statistical reporting (0-1)	Clear variables? (0-2)	Total (Max 13)
Bailey et al 2014	1<br><br>All deaths and all contempt of court for truancy	1<br><br>Denominator = total population	1<br><br>Official records	1<br><br>Yes, outcome was death	0<br><br>None	1<br><br>Official records	1<br><br>6 years	1<br><br>Official records	1<br><br>Univariate	0<br><br>RR and CI no P value	1<br><br>No timescale for truancy	**9**
Borowsky 2001	1<br><br>Random sample of schools. 90,000 of 120,000 completed survey – core random sample selected for interviews (N 13000 is the core sample)	1<br><br>One cohort containing exposed and non-exposed	0<br><br>Self-reported	1<br><br>Outcome was suicide attempt in past 12 months	2<br><br>Not included in multivariate model as non-significant but adjusted for age, gender, family structure and welfare status	1<br><br>Self-reported	1<br><br>Attempts in past 12 months	0<br><br>20,000 at time 1, 14,000 at time 2, no comment on characteristics	1<br><br>>10 EPV (4 variables - event rate>40 for most but not all groups)	1<br><br>OR and P value but no CI 'not enough space, available on request'	1<br><br>12 months suicide attempts clear, how often missed school unclear	**10**
Fergusson 2003	1<br><br>Unselected birth cohort	1<br><br>One cohort containing exposed and non-exposed	0<br><br>Self and parental reports	1<br><br>Since previous assessment	0<br><br>Not clear what is controlled for or if truancy/suspension included or NS in final model	1<br><br>Self-report	1<br><br>Outcomes measured between age 15 and 21, followed up since age 1	1<br><br>84% of the original cohort had data on suicidal behaviour. Sample bias statistically managed	1<br><br>Analysis performed which showed sample bias minimal	1<br><br>B coefficient, SE and P value	1<br><br>Suicidal behaviour unclear over what time period reported in results, suspensions before age 15	**9**
Larsson 2008	1<br><br>Stratified random sampling of schools from 2 counties, 88% response rate	1<br><br>One cohort containing exposed and non-exposed groups	0<br><br>Self-reported	1<br><br>Lifetime at T1 and last 12 months at T2	2<br><br>Attendance not in the model but many covariates were - gender and age were considered univariately but age not in the model (probably due to non-significance) - attendance not in the model	1<br><br>Self-reported	1<br><br>Yes, lifetime and 1 year	0<br><br>4.3% lost to follow up, some differences between these groups – not included in analysis	1<br><br>Univariate	0<br><br>Reported percentages	1<br><br>Not clear which time points was measured at	**9**
Lewinsohn 1994	1	1	0	1	1	1	1	1	1	0	2	**10**

	Randomly selected from representative high schools, only minor differences compared to census data	One cohort containing exposed and non-exposed groups	Self-reported	Suicide attempt between first and second assessments	Gender in the model, not age, lots of others in model but not attendance	Self-reported	Yes one year	88.2% follow up, groups did not differ	>10 EPV - only adjusted for gender	OR NS – no figures given	Both clear, suicidal behaviour between assessments, missed school continuous variable	

Cross sectional studies

Reference	Representativeness of sample (0-1)	Sample size (0-1)	Non-respondents (0-1)	Ascertainment of exposure (0-1)	Comparability - adjustment for confounders (0-2)	Ascertainment of self-harm (0-1)	Statistical reporting (0-1)	Clear variables? (0-1)	Total (Max 10)
Asante 2017	**1**<br><br>Random sample of classes	**1**<br><br>>10 EPV	**0**<br><br>Response rate not stated	**0**<br><br>Self-reported	**2**<br><br>Age, gender and others	**1**<br><br>Self-reported	1<br><br>OR and CI and value	2<br><br>12 months suicidal behaviour and 30 day poor attendance	**8**
Bjarnason 1994	**1**<br><br>Whole population, not sample	**1**<br><br>>10 EPV	**0**<br><br>Number of non-respondents not stated, missing data replaced by group means	**0**<br><br>Self-reported	**2**<br><br>All in one grade, analysis separated by gender) and several other covariates	**1**<br><br>Self-reported	0<br><br>R coefficient and P value only	1<br><br>Lifetime suicide attempts but not clear how info on truancy used in analysis	**6**
Borowsky 1999	0<br><br>20% of eligible population. Convenience sample	1<br><br>Univariate	0<br><br>No comment on differences between those who did (88%) and didn't answer questions on suicide	0<br><br>Self-reported	2<br><br>Age, gender and others are included in the model	1<br><br>Self-reported	0<br><br>Proportions and P value (X2)	2<br><br>Lifetime suicide attempts and truancy binary	**6**
Brunner 2013	1<br><br>Random sample.	1<br><br>>10 EPV	1<br><br>response rate of selected schools 67.5%, 49% consent rate (much worse in 3 of the 11 countries). Study sites 'reasonably' representative of population, missing values imputed	0<br><br>Self-reported	2<br><br>Gender, age and others	1<br><br>Self-reported	1<br><br>OR/RRR and P value	1<br><br>Lifetime direct self-injurious behaviour (clear definition), not clear how truancy measured	**8**
Cheng 2009	1<br><br>Random sample of schools and classes	1<br><br>>10 EPV	1<br><br>98.28% response rate	0<br><br>Self-reported	2<br><br>Age, gender and others	1<br><br>Self-reported	1<br><br>OR, CI and P value	2<br><br>Both clear	**9**

Cwik 2015	0<br><br>Self-selected from Apache surveillance system	1<br><br>Univariate	0<br><br>Missing data left out of analysis	0<br><br>Structured interviews and self-report surveys	0<br><br>None - univariate only	1<br><br>Surveillance system	0<br><br>Percentages and P value? Chi squared, t test, fishers exact test	1<br><br>Single vs multiple suicide attempts in past 90 days, days missed school – not clear what comparisons made	**3**
Davaasambu 2017	1<br><br>Randomised cluster sample from WHO survey	1<br><br>>10 EPV (roughly 24 variables - not clear re event rate for urban and rural separately but likely more than 250)	0<br><br>50 individuals excluded due to missing data - not checked for characteristics	0<br><br>Self-reported survey	2<br><br>Many - school grade not included in multivariate analysis because it was not associated on univariate analysis	1<br><br>Self-reported survey	1<br><br>OR, CI and P value	2<br><br>Clear variables	**8**
Donath et al 2014	1<br><br>Representative sampling framework	1<br><br>>10 EPV (4000 events)	0<br><br>6000 parents refused or absent, 21000 teachers/school refused (62.1%)	0<br><br>Self-reported	2<br><br>Age, gender and others	1<br><br>Self-reported	1<br><br>OR CI and P value	2<br><br>Both clear	**8**
Epstein 2009	1<br><br>Stratified sampling of schools then randomly within schools, nationally representative sample	1<br><br>N = 13917, events not mentioned	0<br><br>School response rate 78%, student 86% (overall response rate 67%). Doesn't say if non respondents differed	0<br><br>Questionnaire	1<br><br>Not age and gender but others included	1<br><br>Questionnaire	1<br><br>OR, CI and P value for suicide attempts	1<br><br>Suicide attempts in past year but skipping school variable not clear	**6**
Evren 2014	1<br><br>Systematic random sampling of 10th grade students in all regions of Turkey (multistage sampling of geographical areas, schools and classes)	1<br><br>Justified	0<br><br>410 excluded due to missing data. Excluded group older and more males	0<br><br>Questionnaire	0<br><br>Age and gender were looked at univariately but then not clear what made it into the model - age, gender and truancy/absenteeism/suspension not mentioned in results of model	1<br><br>Questionnaire	0<br><br>OR CI and P value only for truancy but not for absenteeism or suspension	1<br><br>Self-harm in past year (however intent to die not asked) but no clear time frame for truancy	**4**
Lewinsohn 1993	**1**<br><br>Random sample from 9 schools, representativeness was checked	**1**<br><br>Univariate	**0**<br><br>Participation rate 61% - no comment on characteristics	**0**<br><br>Interview and questionnaire	**2**<br><br>Gender yes, age no due to non-significance on univariate, attendance not included as non-significance but several others included	**1**<br><br>Interview and questionnaire	**1**<br><br>OR, CI and P value	**1**<br><br>Lifetime suicide attempts but days missed school in past 6 weeks not clear how measured	**7**

Pages 2004	1<br><br>Randomly sampled from public and private schools – representative	1<br><br>>10 EPV (only boys in multivariate model)	0<br><br>90% response rate per question – does not explore who did and didn't answer	0<br><br>Questionnaire	2<br><br>Age yes, only boys in multivariate model but yes others	1<br><br>Questionnaire	1<br><br>OR, CI and P value for logistic regression	1<br><br>Suicide attempts, clear. Not clear what comparisons made re absenteeism	**7**
Pillai 2009	1<br><br>All young people in selected areas.	0<br><br><10 EPV (? Entered into model with the combined factors from all the tables which is more than 14?)	1<br><br>A priori analysis, some questions not relevant for some participants. Refusal rate 2.4% rural and 5.2% urban	0<br><br>Interview	2<br><br>Yes, gender age and others	1<br><br>Interview	1<br><br>OR, CI and P value	2<br><br>Both clear – in 3 months	**8**
Randall 2017	1<br><br>Randomly selected classes	1<br><br>>10 EPV	1<br><br>90% response rate	0<br><br>Questionnaire	2<br><br>Gender, age and others	1<br><br>Questionnaire	1<br><br>RR, CI, P value	2<br><br>Ideation/plans/attempts over 12 months, miss school over 30 days	**9**
Sharma 2015	1<br><br>Randomly selected grades, schools and areas from 2 districts	1<br><br>Justified and calculation provided (also >10 EPV - 8 variables in model 1)	0<br><br>Some students missing on the day and others did not complete questions correctly, not accounted for	0<br><br>Questionnaire	2<br><br>Gender but not age (due to non-significance) included in regression model with absenteeism. Yes others	1<br><br>Questionnaire	1<br><br>OR, CI and P value	2<br><br>Suicidal ideation and attempt in past 12 months. Missed school in past 30 days, clear parameters	**8**
Taliaferro 2014	1<br><br>88% of Minnesota districts, 60-80% of students – from Minnesota student survey	1<br><br>>10 EPV	0<br><br>Missing data not mentioned	0<br><br>Survey	2<br><br>Age and others yes, genders analysed separately	1<br><br>Survey	1<br><br>OR, CI and P value	2<br><br>Both clear, lifetime suicidal thoughts/attempts. Missed school in last 30 days	**8**
Xin et al 2017	1<br><br>Random sample	1<br><br>>10 EPV (3563 events)	1<br><br>90% response rate	0<br><br>Self-reported	2<br><br>Age, gender and others	1<br><br>Self-reported	1<br><br>OR, CI and P value	1<br><br>NSSI clear, truancy not clear	**8**

Case control studies

Reference	Case definition (0-1)	Representativeness (0-1)	Selection of controls (0-1)	Definition of controls (0-1)	Comparability - adjustment for confounders (0-2)	Ascertainment of exposure (0-1)	Same method of ascertainment for cases and controls (0-1)	Non-response rate (0-1)	Sample size (0-1)	Statistical reporting (0-1)	Clear variables? (0-2)	Total (Max 13)
Lyon et al 2000	1<br><br>Presentations to hospital with suicide attempts	1<br><br>All presentations	1<br><br>Walk-ins to same department	1<br><br>No history of suicide attempt	2<br><br>Matched gender and age, controlled for others	0<br><br>Self-report	1<br><br>Yes	1<br><br>Incomplete data for 2 cases and 2 controls	0<br><br>38 cases, at least 10 covariates, <10 EPV	0<br><br>Should have done conditional logistic regression	1<br><br>Suicide attempts yes, truancy not clear	**9**
Noble et al 2011	1<br><br>Self-report	1<br><br>Random sample	1<br><br>Community controls	1<br><br>No history of NSSI	2<br><br>Matched gender and age, controlled for others	0<br><br>Self-report	1<br><br>Yes	0<br><br>Not reported	1<br><br>638 outcomes, >10 EPV	0<br><br>No CI	2<br><br>Both clear	**10**

PRISMA and MOOSE checklists

PRISMA

Section/topic	#	Checklist item	Reported on page #
**TITLE**			
Title	1	Identify the report as a systematic review, meta-analysis, or both.	1
**ABSTRACT**			
Structured summary	2	Provide a structured summary including, as applicable: background; objectives; data sources; study eligibility criteria, participants, and interventions; study appraisal and synthesis methods; results; limitations; conclusions and implications of key findings; systematic review registration number.	1
**INTRODUCTION**			
Rationale	3	Describe the rationale for the review in the context of what is already known.	1-2
Objectives	4	Provide an explicit statement of questions being addressed with reference to participants, interventions, comparisons, outcomes, and study design (PICOS).	3
**METHODS**			
Protocol and registration	5	Indicate if a review protocol exists, if and where it can be accessed (e.g., Web address), and, if available, provide registration information including registration number.	3
Eligibility criteria	6	Specify study characteristics (e.g., PICOS, length of follow-up) and report characteristics (e.g., years considered, language, publication status) used as criteria for eligibility, giving rationale.	3-4
Information sources	7	Describe all information sources (e.g., databases with dates of coverage, contact with study authors to identify additional studies) in the search and date last searched.	3
Search	8	Present full electronic search strategy for at least one database, including any limits used, such that it could be repeated.	Supplement
Study selection	9	State the process for selecting studies (i.e., screening, eligibility, included in systematic review, and, if applicable, included in the meta-analysis).	3
Data collection process	10	Describe method of data extraction from reports (e.g., piloted forms, independently, in duplicate) and any processes for obtaining and confirming data from investigators.	4
Data items	11	List and define all variables for which data were sought (e.g., PICOS, funding sources) and any assumptions and simplifications made.	3-4 & Supplement
Risk of bias in individual studies	12	Describe methods used for assessing risk of bias of individual studies (including specification of whether this was done at the study or outcome level), and how this information is to be used in any data synthesis.	4

Summary measures	13	State the principal summary measures (e.g., risk ratio, difference in means).	4
Synthesis of results	14	Describe the methods of handling data and combining results of studies, if done, including measures of consistency (e.g., I^2) for each meta-analysis.	4

Section/topic	#	Checklist item	Reported on page #
Risk of bias across studies	15	Specify any assessment of risk of bias that may affect the cumulative evidence (e.g., publication bias, selective reporting within studies).	4
Additional analyses	16	Describe methods of additional analyses (e.g., sensitivity or subgroup analyses, meta-regression), if done, indicating which were pre-specified.	4
**RESULTS**			
Study selection	17	Give numbers of studies screened, assessed for eligibility, and included in the review, with reasons for exclusions at each stage, ideally with a flow diagram.	4
Study characteristics	18	For each study, present characteristics for which data were extracted (e.g., study size, PICOS, follow-up period) and provide the citations.	Table 1 & Supplement
Risk of bias within studies	19	Present data on risk of bias of each study and, if available, any outcome level assessment (see item 12).	5 and Supplement
Results of individual studies	20	For all outcomes considered (benefits or harms), present, for each study: (a) simple summary data for each intervention group (b) effect estimates and confidence intervals, ideally with a forest plot.	5-8 and Tables 2&3
Synthesis of results	21	Present results of each meta-analysis done, including confidence intervals and measures of consistency.	5-8
Risk of bias across studies	22	Present results of any assessment of risk of bias across studies (see Item 15).	6,8 & Supplement
Additional analysis	23	Give results of additional analyses, if done (e.g., sensitivity or subgroup analyses, meta-regression [see Item 16]).	6 & 8
**DISCUSSION**			
Summary of evidence	24	Summarize the main findings including the strength of evidence for each main outcome; consider their relevance to key groups (e.g., healthcare providers, users, and policy makers).	8-9
Limitations	25	Discuss limitations at study and outcome level (e.g., risk of bias), and at review-level (e.g., incomplete retrieval of identified research, reporting bias).	9-10
Conclusions	26	Provide a general interpretation of the results in the context of other evidence, and implications for future research.	11
**FUNDING**			
Funding	27	Describe sources of funding for the systematic review and other support (e.g., supply of data); role of funders for the systematic review.	11

1. Meta-analysis of Observational Studies in Epidemiology (MOOSE) checklist

Item No	Recommendation	Reported on Page No
Reporting of background should include		
1	Problem definition	2
2	Hypothesis statement	2
3	Description of study outcome(s)	3-4
4	Type of exposure or intervention used	3
5	Type of study designs used	3
6	Study population	3
Reporting of search strategy should include		
7	Qualifications of searchers (eg. librarians and investigators)	3
8	Search strategy, including time period included in the synthesis and key words	Supplement
9	Effort to include all available studies, including contact with authors	3
10	Databases and registries searched	3
11	Search software used, name and version, including special features used (eg, explosion)	Supplement
12	Use of hand searching (eg, reference lists of obtained articles)	3
13	List of citations located and those excluded, including justification	Supplement
14	Method of addressing articles published in languages other than English	N/A
15	Method of handling abstracts and unpublished studies	3
16	Description of any contact with authors	N/A
Reporting of methods should include		

17	Description of relevance or appropriateness of studies assembled for assessing the hypothesis to be tested	3-4
18	Rationale for the selection and coding of data (eg, sound clinical principles or convenience)	3-4
19	Documentation of how data were classified and coded (eg, multiple raters, blinding and interrater reliability)	4
20	Assessment of confounding (eg, comparability of cases and controls in studies where appropriate)	4
21	Assessment of study quality, including blinding of quality assessors, stratification or regression on possible predictors of study results	4
22	Assessment of heterogeneity	4
23	Description of statistical methods (eg, complete description of fixed or random effects models, justification of whether the chosen models account for predictors of study results, dose-response models, or cumulative meta-analysis) in sufficient detail to be replicated	4
24	Provision of appropriate tables and graphics	Figures 1-3, Tables 1-3 & Supplement
Reporting of results should include		
25	Graphic summarizing individual study estimates and overall estimate	Figure 2 & 3
26	Table giving descriptive information for each study included	Table 1
27	Results of sensitivity testing (eg, subgroup analysis)	6 & 8
28	Indication of statistical uncertainty of findings	Throughout
